# Comprehensive quantification of C4 to C26 free fatty acids using a supercritical fluid chromatography-mass spectrometry method in pharmaceutical-grade egg yolk powders intended for total parenteral nutrition use

**DOI:** 10.1007/s00216-025-05732-3

**Published:** 2025-01-23

**Authors:** Mark Dennis Chico Retrato, Anh Vu Nguyen, S. J. Kumari A. Ubhayasekera, Jonas Bergquist

**Affiliations:** Department of Chemistry – Biomedical Center, Analytical Chemistry and Neurochemistry, Uppsala University, Uppsala, Sweden

**Keywords:** SFC-MS, FFA analysis, Analytical method development, Pharmaceutical-grade egg yolk powders, Total parenteral nutrition

## Abstract

Free fatty acids (FFAs) are important energy sources and significant for energy transport in the body. They also play a crucial role in cellular oxidative stress responses, following cell membrane depolarization, making accurate quantification of FFAs essential. This study presents a novel supercritical fluid chromatography-mass spectrometry (SFC-MS) method using selected ion recording in negative electrospray ionization mode, enabling rapid quantification of 31 FFAs within 6 min without derivatization. FFAs are identified and quantified using an HSS C18 SB column and a secondary mobile phase consisting of methanol with formic acid by detecting their [M − H]^−^ ions. Calibration curves showed strong linearity (*R*^2^ ≥ 0.9910), spanning 1000–12,000 ng/mL for short-chain FFAs and 50–1200 ng/mL for medium- and long-chain FFAs. The method achieves detection limits as low as 1 ng/µL for short-chain FFAs and 0.05 pg/µL for other FFAs per on-column injection. The method demonstrated high accuracy and precision, with bias and coefficients of variation maintained below 15% across five quality control levels. Freeze–thaw and autosampler stability studies confirmed the behavior of matrix-matched standards under optimal storage conditions. The validated method was applied to the analysis of pharmaceutical-grade egg yolk powders, using 13 deuterated FFAs as internal standards (IS) in comparison with heptadecanoic acid (C17:0). Significant variations in FFA quantification using two different IS approaches underscore the importance of selecting an appropriate IS. In summary, this study introduces a reliable and validated SFC-MS method for analyzing FFAs ranging from C4 to C26, requiring minimal sample preparation.

**Graphical Abstract:**

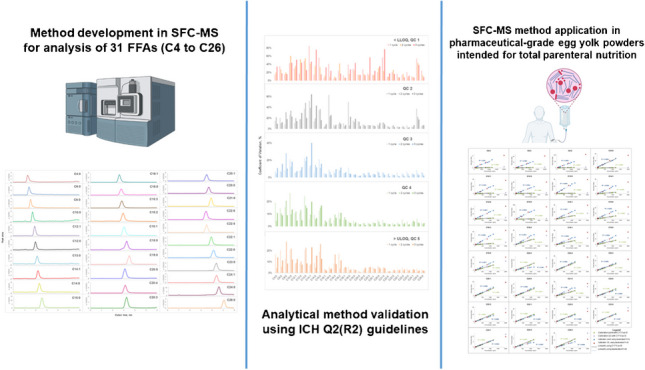

**Supplementary Information:**

The online version contains supplementary material available at 10.1007/s00216-025-05732-3.

## Introduction

Free fatty acids (FFAs) are unesterified fatty acids present in pharmaceutical lipid emulsions (PLEs) intended for total parenteral nutrition (TPN). Fatty acids (FAs) are typically found in tissues or the bloodstream as components of complex lipids, like triglycerides and phospholipids, while FFAs circulate in their unbound, free form. Hydrolysis of many dietary complex lipids into FFAs occurs before their absorption and further utilization in lipid synthesis [1]. Maintaining an appropriate balance of FFAs in PLEs is essential for ensuring nutritional adequacy, as FFAs play a critical role in energy production [2, 3]. Moreover, FFAs contribute to cellular functions and modulate inflammatory responses and immune challenges [2–5]. In PLE formulations, FFA levels are carefully monitored, as compositional changes can yield both beneficial and adverse health effects in clinical subjects. Evaluating the FFA content provides valuable insights into recovery and therapeutic outcomes in critically ill patients receiving TPN [6–9]. PLEs degrade over time, and tracking FFA content over extended periods can help detect emulsion rancidity. Therefore, assessing FFAs in PLEs is crucial for product safety and efficacy, as TPN products must meet strict quality and regulatory standards [6, 8].

There is growing interest in the comprehensive analysis of FFAs, given their diverse roles in health, including energy production, cellular signaling, metabolic regulation, and immune modulation [10–14]. Different kinds of FFAs, from short-chain to long-chain, are known to affect many bodily functions. Some FFAs have shown promise in disease prevention, like their ability to fight cancer and inflammation [15, 16]. Several analytical approaches, primarily liquid chromatography (LC) and gas chromatography (GC) have been used to quantify FFAs across a broad range in a single method [17–20]. While established methods exist for quantifying medium- and long-chain free fatty acids (MCFFAs and LCFFAs), there are technical challenges in incorporating short-chain free fatty acids (SCFFAs) into the same method. Our recent study quantified 22 FFAs from C6 to C24 using GC–MS; however, this approach requires transesterification of FFAs to fatty acid methyl esters (FAMEs) for GC compatibility. Five different chemical derivatization methods were employed, with the percent yield for each method being lower than 50% [17]. Ideally, FFAs should be analyzed in their native form, but derivatization techniques for GC analysis are not fully efficient, even with longer reaction times ranging from 30 min to over 24 h [21]. LC-based methods face similar limitations, as they also require derivatization. For example, an LC–MS method developed to analyze FFAs from C2 to C24 relied on twin derivatization agents, 5-(dimethylamino) naphthalene-1-sulfonylpiperazine and (diethylamino)naphthalene-1-sulfonyl piper-azine as derivatization agents [18]. Other derivatization strategies used for quantifying SCFFAs include 3-nitrophenylhydrazine, *O*-benzyl hydroxylamine, and 2-picolylamine [22, 23]. These studies highlight the difficulty of analyzing FFAs via LC- and GC–MS due to inherent technical limitations. In addition, detection of SCFFAs alongside other FFAs is challenging due to their instability, volatility, and low concentrations [17, 24–26]. The limited options for column chemistry, along with the need for chemical derivatization and extensive sample preparation, make the quantification of SCFFAs and other FFAs difficult [17, 25]. Most current LC- and GC–MS techniques for quantifying FFAs span from 12 to 60 min [17, 18, 21, 22, 24–28]. Therefore, there is a need to develop a steadfast, robust, and straightforward method for analyzing a broader range of FFAs.

Supercritical fluid chromatography (SFC) has emerged as a valuable technique for comprehensive lipid analysis [4, 29, 30]. When applied to the quantification of FFAs, SFC offers distinct advantages over traditional methods, including eliminating the need for chemical derivatization and the ability to couple with a wide range of detectors. Early stages of FFA analysis without derivatization in SFC was accomplished using various chemical detectors, such as flame ionization (FID) [31], Fourier transform infrared (FT-IR) [32], and ultraviolet (UV) [33]. These tandem techniques produced irregular peak shapes, longer analysis time, and necessitating pre-analysis handling [20]. These methods had difficulties in reproducibility, and usually preparative thin-layer chromatography (TLC) is required to achieve optimum analyte resolution [20]. Hence, there is a need for a more sensitive detector in developing a more comprehensive method that can address these limitations. Coupling SFC with a more sophisticated detector such as mass spectrometry (MS) was done recently for FFA quantification [34–36]. With supercritical fluids as the primary mobile phase, SFC-MS methods provide exceptional efficiency and speed, supporting high-throughput analyses [4, 29]. The flexibility in column selection and the use of organic co-solvents further enhance the separation of FFAs with different polarities. Furthermore, SFC requires only small sample volumes and significantly reduces organic solvent consumption, making it an environmentally friendly option by producing less organic waste compared to other MS-based separation techniques [29, 30]. These features make SFC highly suitable for rapid, efficient, and sustainable FFA quantification across a wide range of chain lengths.

This study presents the development and validation of a novel supercritical fluid chromatography-mass spectrometry (SFC-MS) method that addresses the limitations of existing methods in the literature. This method for the first time enables the quantification of 31 saturated and unsaturated FFAs, covering a broad range of chain lengths from C4 to C26 without any chemical derivatization within an efficient 6-min run time. The validated SFC-MS method was applied to pharmaceutical-grade egg yolk powders, a key component of PLEs used in TPN manufacturing. Additionally, this study provides a comprehensive comparison of FFA quantification using deuterated FFAs against margaric acid (C17:0) as internal standards in matrix-matched standards and samples.

## Materials and methods

### Standards and reagents

Stock FFA standards (C4:0 to C26:0) at concentrations of 1, 2, or 10 mg/mL were prepared in chloroform and stored at − 20 °C since 2013. Deuterated internal standards (IS) C4:0-*d*_7_, C10:0-*d*_19_, C12:0-*d*_23_, C14:0-*d*_27_, C16:0-*d*_31_, C16:1-*d*_14_, C18:0-*d*_35_, C18:1-*d*_17_, C18:2-*d*_4_, C18:3-*d*_5_, C20:4-*d*_11_, C20:5-*d*_5_, and C22:6-*d*_5_ were purchased from Larodan AB (Stockholm, Sweden). MS-grade methanol (MeOH), isopropanol (IPA), hydrochloric acid (HCl), ammonium formate (NH_4_COOH), ammonium fluoride (NH_4_F), ammonium hydroxide (NH_4_OH), and formic acid (FA) were purchased from Fisher Scientific (Göteborg, Sweden). Ultrapure water (MilliQ, Merck, Stockholm, Sweden) was used in all preparations and dilutions as needed. Pharmaceutical-grade egg yolk powders were obtained from a drug manufacturing company (Uppsala, Sweden).

### Instrumentation

Analytical separation of the FFA standards was performed by ultra-performance supercritical fluid chromatography (UPSFC) on an Acquity UPC^2^ system (Waters Corporation, Milford, USA). Supercritical CO_2_ (≥ 99.998%, Air Liquide AB, Sweden) served as mobile phase A, while MeOH with 0.1% formic acid was used as mobile phase B. Instrument back pressure was regulated at 2000 psi with the sample chamber held at 10 ± 2 °C, and an injection volume of 2.0 µL. Method optimization involved screening two columns: the Torus 1-AA column (130 Å, 1.7 µm, 2.1 × 100 mm) and HSS C18 SB column, (100 Å, 1.8 µm, 3.0 × 100 mm) (Waters Corporation, Milford, USA). The optimized method employed the HSS C18 column, kept at 50 °C during analysis. A gradient elution was applied, starting from 95% sCO_2_ held at 1 min, then slowly going to 80% sCO_2_ at 4 min, then finishing the analytical run at 6 min with 95% sCO_2_. These time points had a constant flow rate of 0.5 mL/min. A clean-up step at 95% sCO_2_ at 0.5 mL/min for 4 min was added to prevent carryover. Methanol with 0.1% NH_4_OH was used as a make-up solvent at a flow rate of 0.2 mL/min.

Mass spectrometric detection was performed on a Xevo TQ-S triple quadrupole mass spectrometer (Waters Corporation, Milford, USA) with electrospray ionization in the negative mode (ESI^−^). The following conditions were applied: capillary voltage of 2.5 kV, source temperature of 110 °C, and cone gas flow rate 150 L/h. Nitrogen was used as the desolvation gas at a temperature of 500 °C and a flow rate of 600 L/h. Argon was used as the collision gas with a flow rate of 0.18 L/h. The optimal cone voltage for each FFAs was determined by injecting 1 µL of each standard at 100 ng/mL except C4:0, C6:0, and C8:0, which were injected at 1000 ng/mL (Table 1). FFA identification was achieved using selected ion recording (SIR) in MassLynx™ 4.1 software (Waters Corporation, Milford, USA), using [M − H]^−^ ions for each FFA.

### FFA standard preparation

Thirty-one (31) different FFA standards were prepared individually at 10,000 µg/mL in methanol. Likewise, thirteen (13) deuterated FFAs and C17:0, which served as IS, were prepared separately in methanol. The lists of analytes (Table S1) and their corresponding IS (Table S2) are indicated. The prepared working solutions were kept at − 20 °C until further analysis.

### Preparation of FFA-free matrix

Analyte-free samples were prepared by treating a commercial egg yolk powder with activated charcoal, followed by removal of the charcoal through centrifugation or filtration [27]. Analyte removal was verified by analyzing a small aliquot of the treated matrix to ensure target FFAs were undetectable, and FFA-free matrices were stored at − 20 °C. Analyte-free samples were used to prepare calibration and quality control samples by spiking them with different concentrations of FFAs.

### Analytical method validation

The optimized SFC-MS method for the analysis of 31 FFAs in a single analytical run was validated in compliance with ICH Q2(R2) guidelines [37], incorporating the standards of the European Medicines Agency (EMA) and the US Food and Drug Administration (FDA) for establishing analytical procedures.

#### Linearity

The calibration curve constructions were performed under two different conditions to evaluate the method. In the first condition, nine calibrators were prepared for most FFA standards at concentrations ranging from 50 to 1200 ng/mL, while C4:0, C6:0, and C8:0 were prepared from 1000 to 12,000 ng/mL. All of these calibrators were prepared in methanol, along with deuterated FFAs as IS included at 100 ng/mL, except for C4:0-*d*_7_ (2000 ng/mL). The second condition used similar concentrations for the FFA standards and IS, but FFA-free matrix was used as the diluent for each calibration point. Blank and zero samples (blank spiked with IS) were prepared for each condition. Standard curves were generated by plotting the analyte concentrations against the ratios of the FFA standard to IS peak areas. Six calibration curves were generated for each analyte across three different days for both conditions. Linearity was evaluated using weighted (1/x) linear regression, and correlation coefficients (*R*^2^) and residual plots were derived from both solvent-based and matrix-matched calibration curves for each FFA using the TargetLynx™ feature of MassLynx™ 4.1 software (Waters Corporation, Milford, USA).

#### Accuracy and precision

Method accuracy and precision were evaluated using quality control (QC) samples (*n* = 6) at five concentration levels. For C4:0, C6:0, and C8:0, the QC concentrations were 500, 1250, 4000, 8000, and 20,000 ng/mL, while for the remaining FFA standards, the QC concentrations were 25, 75, 350, 800, and 2000 ng/mL. Accuracy was expressed as bias and assessed at five QC levels (*n* = 6) over three different days. Precision was evaluated using the coefficient of variation (CV), with intraday measurements (*n* = 6) and interday measurements across three different days (*n* = 6), at all QC levels. The FFA concentrations were measured using the IS method applied in linearity performance tests.

#### Spiking and recovery studies

Method recovery was measured by spiking the FFA-free matrix (*n* = 6) with C4:0, C6:0, and C8:0 at a final concentration of 1000 ng/mL, while the remaining FFA standards were spiked at a concentration of 100 ng/mL. Similarly, the deuterated FFA IS (each at 100 ng/mL), except for C4:0-*d*_7_ (2000 ng/mL), were added to each recovery setup. Six measurements were performed per prepared sample. Recovery was assessed by comparing the mean spiked concentrations against the mean unspiked concentrations.

#### Carryover

Systematic carryover testing during method development was accomplished by analyzing a solvent blank following the injection of the highest calibration standard (C4:0, C6:0, and C8:0 at 12,000 ng/mL; the rest of the FFA standards at 1200 ng/mL). The analyte peak areas in the solvent blank were compared to those at the lower limit of quantification (LLOQ).

#### Freeze–thaw and ambient temperature stability measurements

The freeze–thaw cycle of the FFA standards was evaluated at five QC levels. For C4:0, C6:0, and C8:0, the QC concentrations were 500, 1250, 4000, 8000, and 20,000 ng/mL, while for the remaining FFA standards, the QC concentrations were 25, 75, 350, 800, and 2000 ng/mL. QC samples stored at − 20 °C were removed from the freezer and kept at room temperature for 60 min prior to analysis. Each QC level (*n* = 6) was analyzed using the developed SFC-MS method and returned to the freezer. This entire process, including the SFC-MS analyses, was repeated twice at 24-h intervals, resulting in three full freeze–thaw cycles for a complete evaluation.

Autosampler stability of the FFA standards was assessed at five QC levels. The same QC concentration levels were applied for C4:0, C6:0, and C8:0, as well as for the remaining FFA standards from the freeze–thaw stability studies. The QC samples stored at − 20 °C were removed from the freezer and kept in the SFC-MS autosampler for 24 h prior to analysis, maintained at 10 °C. Each QC level (*n* = 6) was analyzed using the developed SFC-MS method. After the first analysis, subsequent measurements were repeated twice at 24-h intervals, corresponding to three full instrument runs over three consecutive days.

### FFA acid extraction and analysis of pharmaceutical-grade egg yolk powders

Pharmaceutical-grade egg yolk powders (*n* = 3) from distinct sources were weighed to the nearest 1 mg. To each sample, 3 mL of IPA:H_2_O:1 M HCl (40:10:1, %v/v) with 0.05 mg/mL butylhydroxytoluene (BHT) as an antioxidant was added, and the mixture was shaken for 24 h. After centrifugation at 10,000 rpm for 30 min at 10 °C, the supernatant was transferred into 15-mL tubes, and 4 mL each of heptane and water were added. The mixture was vortexed for 10 min and centrifuged at 12,000 rpm at 8 °C for 30 min. The upper heptane layer was collected, and the extraction was repeated with an additional 4 mL of heptane. The heptane layers were pooled and dried under a gentle stream of nitrogen gas. The residues were reconstituted in 200 µL methanol, to which 50 µL of IS mixture containing C4:0-*d*_7_ (2000 ng/mL), other deuterated FFAs-IS (100 ng/mL), and C17:0 (200 ng/mL) was added. Samples were filtered through 0.22-µm nylon filters, transferred to vials, and analyzed in triplicate using the optimized SFC-MS method. Data were processed using TargetLynx™ (Waters Corporation, Milford, USA).

## Results and discussion

Free fatty acids (FFAs) are a fundamental class of lipids with significant roles in metabolism, cellular signaling, and clinical diagnostics, making their accurate and precise measurement essential. However, analysis of FFAs with a broad range of chain lengths and degrees of unsaturation in a single analytical method is challenging. Selecting an optimal chromatographic approach for FFAs quantification requires careful consideration of the physical and chemical properties of individual FFAs, such as stability, polarity, isomeric complexity, low concentrations, and volatility. This study presents a novel SFC-MS method that overcomes key limitations associated with traditional GC and LC techniques for FFA analysis. Unlike GC and LC, which often require derivatization and extensive sample preparation, SFC-MS enables the direct, efficient quantification of FFAs in their native form. This method allows for high-throughput analysis across a wide range of FFAs chain lengths (C4 to C26) within 6 min, offering both sensitivity and environmental benefits through reduced organic solvent use. Our comprehensive method development in SFC-MS was thoroughly validated according to the ICH Q2(R2) guidelines used in targeted analysis for pharmaceutical and clinical applications, and this work is first one reported to best of our knowledge. Significant emphasis is given on the modified Dole extraction method optimized in-house for reproducible recovery and reliable quantification of FFAs. This method utilized a straightforward solvent phase extraction without any enrichment procedures or clean-up. In summary, our SFC-MS method increased analytical performance of comprehensive FFA quantification by cutting down chromatographic run times and sample preparation while increasing analyte sensitivity.

**Table 1:** Summary of chemical properties and SFC-MS parameters optimized for 31 FFA standards and 14 FFA internal standards

	Compound	logP	pKa	RT, min	[M-H]^−^, m/z	CV (V)
1	C4:0	0.79	4.84	1.33	87.1	45
2	C6:0	4.09	4.90	1.43	115.1	45
3	C8:0	3.05	4.89	1.59	143.1	45
4	C10:0	4.90	4.09	1.76	171.2	30
5	C12:1	2.96	4.72	1.94	197.2	30
6	C12:0	4.60	5.30	2.03	199.2	30
7	C13:0	5.57	4.95	2.21	213.2	30
8	C14:1	5.38	4.99	2.27	225.2	30
9	C14:0	5.37	4.90	2.37	227.2	30
10	C15:0	6.62	4.95	2.58	241.2	30
11	C16:1	6.40	4.99	2.62	253.2	30
12	C16:0	7.17	4.75	2.80	255.2	30
13	C18:3	6.46	4.99	2.83	277.2	30
14	C18:2	7.18	4.77	2.95	279.2	30
15	C18:1	7.70	4.99	3.05	281.3	30
16	C18:0	8.23	4.50	3.30	283.3	30
17	C19:0	8.75	4.95	3.52	297.3	30
18	C20:5	6.23	4.82	3.12	301.2	30
19	C20:4	6.91	4.82	3.16	303.2	30
20	C20:3	7.56	4.89	3.28	305.3	30
21	C20:1	8.40	4.95	3.55	309.3	30
22	C20:0	8.53	4.95	3.73	311.3	30
23	C21:0	9.81	4.95	3.94	325.3	30
24	C22:6	6.78	4.89	3.49	327.2	30
25	C22:5	7.26	4.96	3.53	329.3	30
26	C22:1	9.82	4.73	3.96	337.3	30
27	C22:0	10.34	4.73	4.18	339.3	30
28	C23:0	10.87	4.95	4.41	353.4	30
29	C24:1	10.89	4.95	4.43	365.4	30
30	C24:0	11.40	4.95	4.65	367.4	30
31	C26:0	12.47	4.95	5.10	395.4	30
*Internal standard*						
A	C4:0-*d*_7_			1.33	94.1	45
B	C10:0-*d*_19_			1.76	190.2	30
C	C12:0-*d*_23_			2.03	222.3	30
D	C14:0-*d*_27_			2.37	254.4	30
E	C16:1-*d*_14_			2.62	267.3	30
F	C16:0-*d*_31_			2.80	286.4	30
G	C18:0-*d*_35_			3.30	318.5	30
H	C18:1-*d*_17_			3.05	298.4	30
I	C18:2-*d*_4_			2.95	283.8	30
J	C18:3-*d*_5_			2.83	284.2	30
K	C20:4-*d*_11_			3.16	314.2	30
L	C20:5-*d*_5_			3.12	306.2	30
M	C22:6-*d*_5_			3.49	332.5	30
N	C17:0	7.68	4.95	3.03	269.3	30

*logP*, logarithm of partition coefficient; *pKa*, negative logarithm of acid dissociation constant; *RT*, retention time; *CV*, cone voltage

### Method development and optimization

**Fig. 1:**
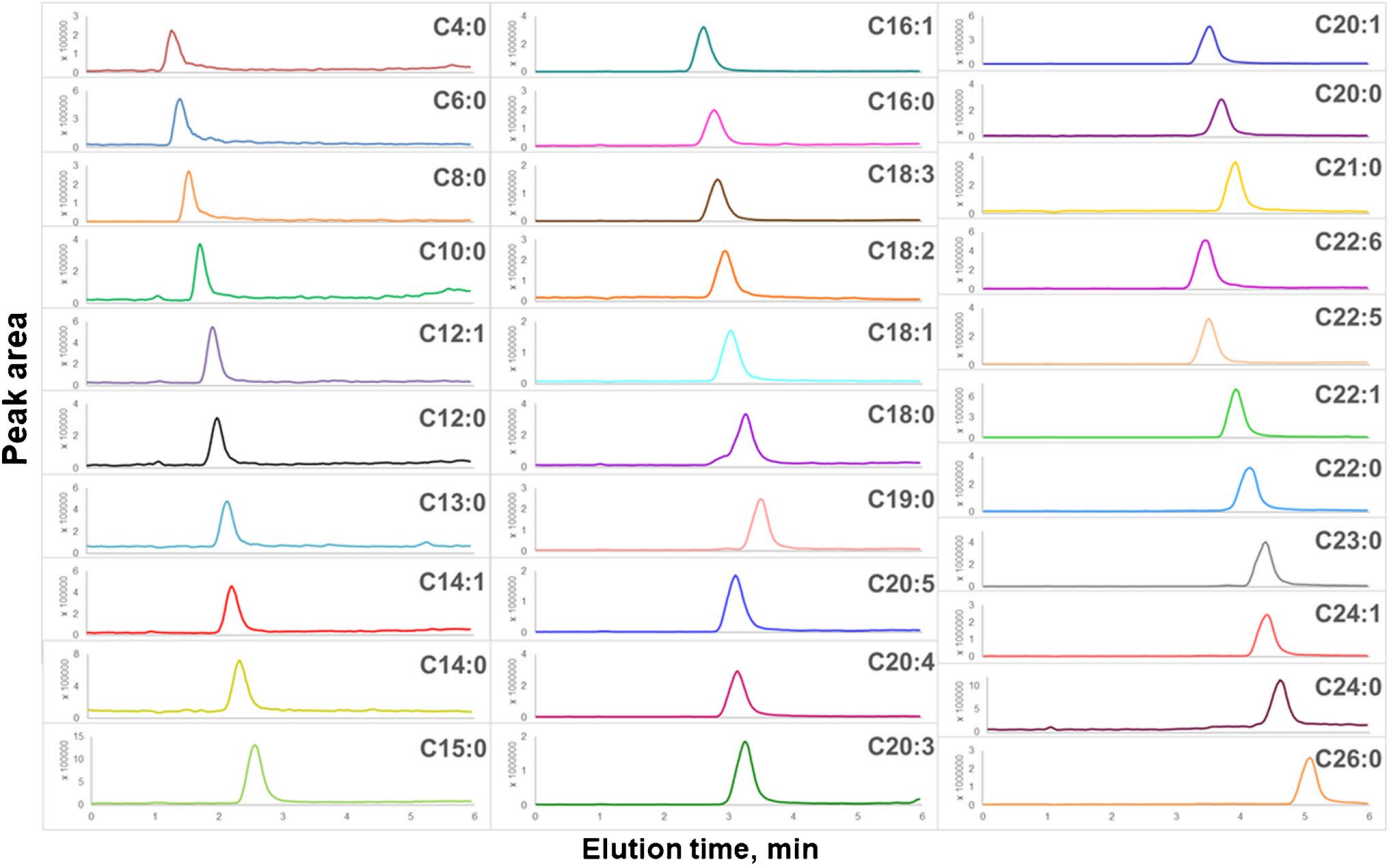
Elution profile of 31 FFA standards using ESI^−^ in the optimized SFC-MS method within an analytical run time of 6 min

**Fig. 2:**
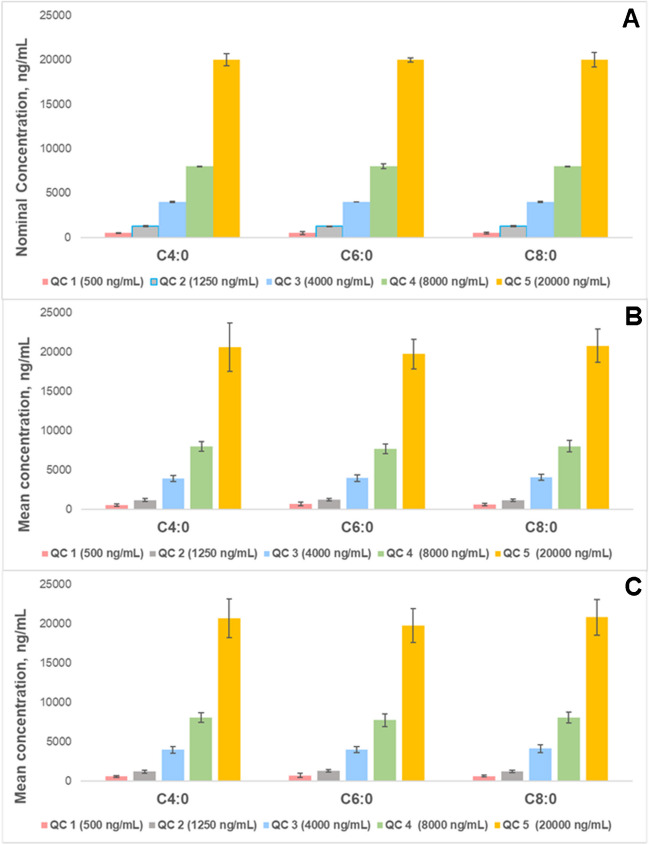
Method performance charts for **A** accuracy expressed as mean concentrations ± bias, ng/mL, **B** intraday precision, and **C** interday precision expressed as mean concentrations ± SD, ng/mL (*n* = 6), of matrix-matched SCFFA standards at five QC concentration levels

**Fig. 3:**
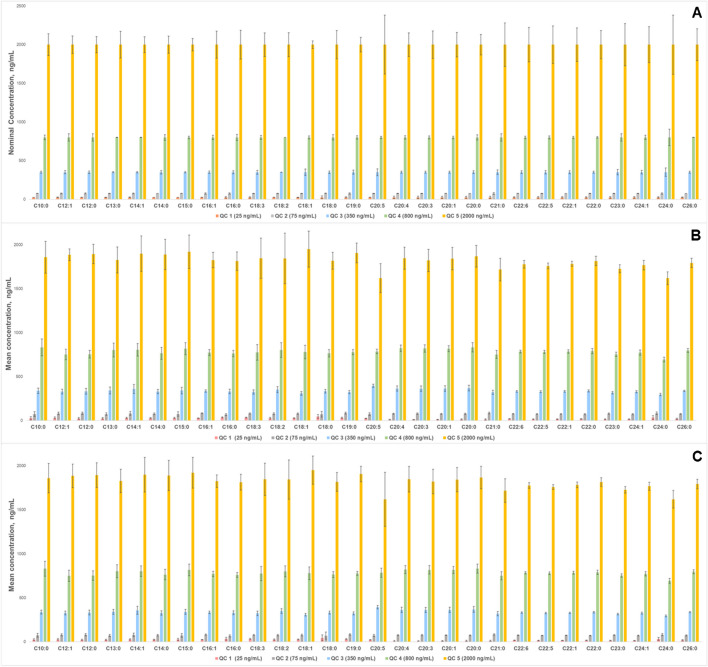
Method performance charts for **A** accuracy expressed as mean concentrations ± bias, ng/mL, **B** intraday precision, and **C** interday precision expressed as mean concentrations ± SD, ng/mL (*n* = 6), of matrix-matched FFA standards from C10:0 to C26:0 at five QC concentration levels

**Fig. 4:**
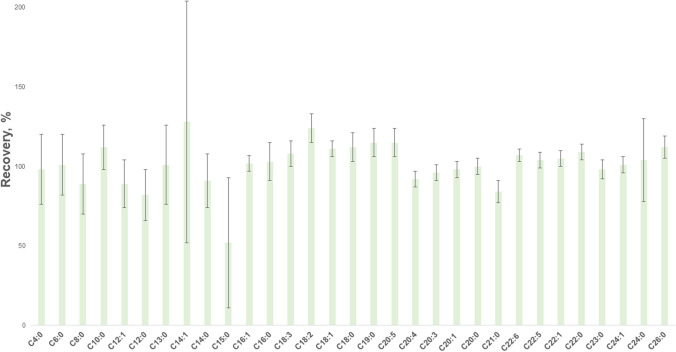
Recovery measurements (*n* = 6) for 31 FFA standards in the validated SFC-MS method

**Fig. 5:**
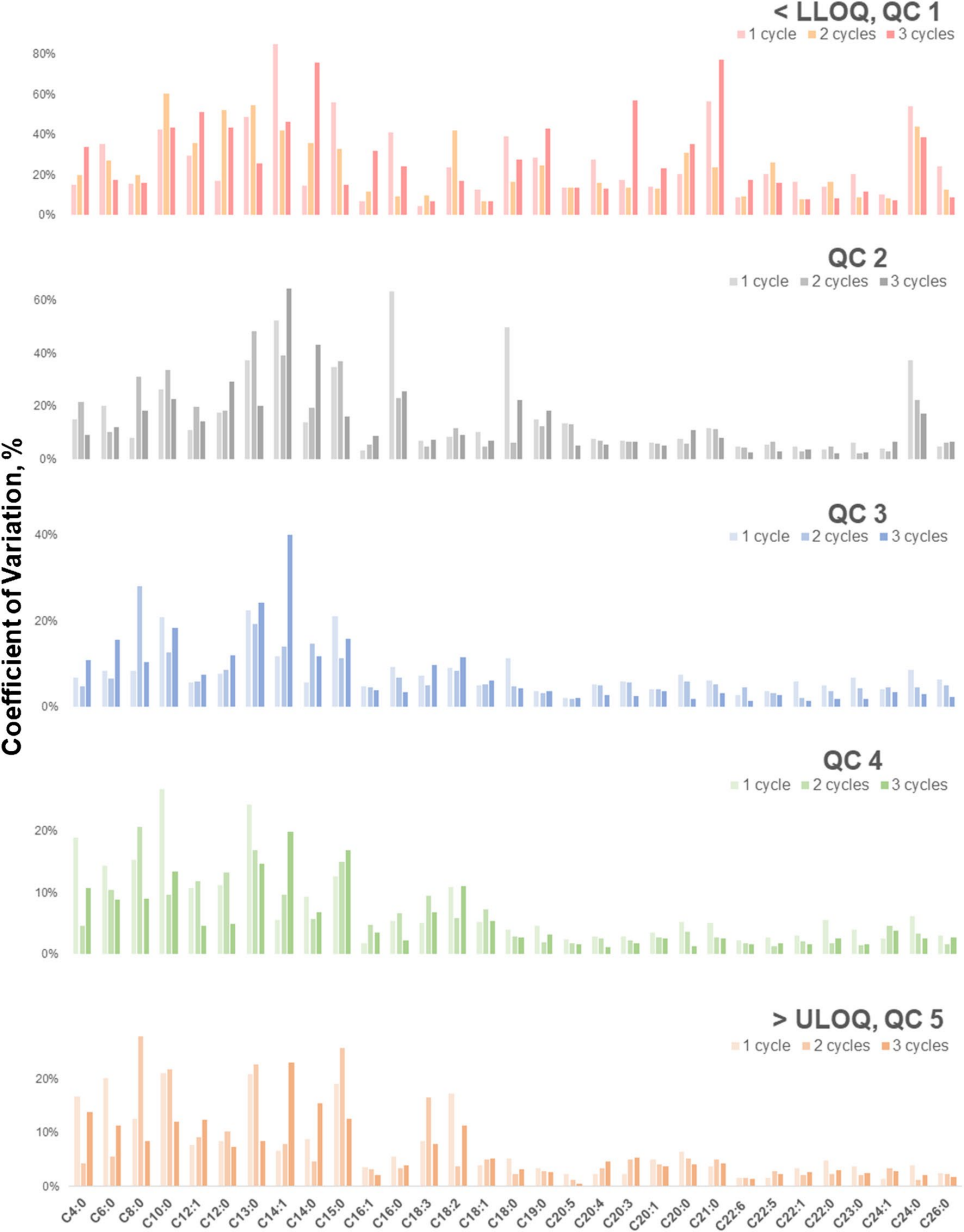
Measurement variabilities in 31 FFA standards (*n* = 6) for assessing freeze–thaw stability of 31 FFAs at five QC concentration levels in the developed SFC-MS method expressed as %CV

**Fig. 6:**
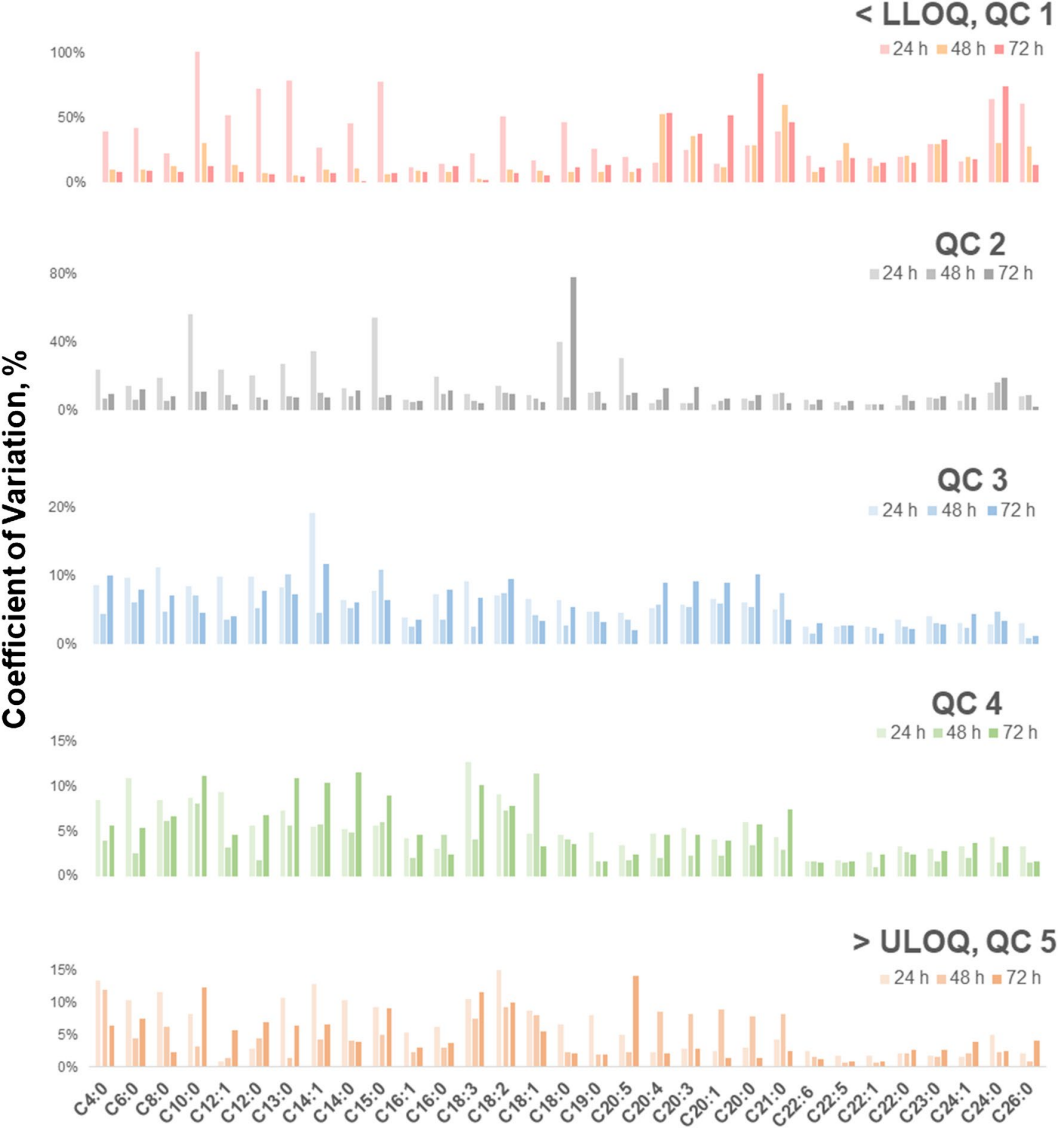
Measurement variabilities in 31 FFA standards (*n* = 6) for assessing autosampler stability of 31 FFAs at five QC concentration levels in the developed SFC-MS method expressed as %CV

This study presents a validated SFC-MS method for the rapid quantification of 31 targeted FFAs in pharmaceutical-grade egg yolk powders used in PLEs for TPN products (Fig. 1). The method, optimized for analysis within 6 min, was validated according to ICH Q2(R2) guidelines (Figs. 2, 3, 4, 5, and 6). FFAs, with their carboxylic acid group, readily generate intense and abundant deprotonated ions ([M − H]⁻) during negative electrospray ionization (ESI^−^) [38]. While multiple reaction monitoring (MRM) is typically used for targeted quantification, selected ion recording (SIR) was chosen for enhanced selectivity. Generating fragment ions such as [M − H – H_2_O]⁻, [M − H − 2H_2_O]⁻, and [M − H − 2H_2_O – CO_2_]⁻ can be challenging [38], and SIR dwell times were optimized to capture at least 12 data points per peak ensuring reproducible peak acquisition. Moreover, the ionization of free fatty acids in negative ESI mode depends on the removal of a proton from the carboxylic group, which is influenced by the fatty acid’s chain length and acidity. In fact, poor ionization efficiency for SCFFAs is observed due to their high pKa and volatility. The low solubility of LCFFAs can reduce ionization efficiency in negative ESI mode. However, this can be improved by optimizing the solvent system with additives like formic acid and adjusting source conditions, such as voltage and gas flow. These changes enhance FFA separation and overall performance in negative ESI mode. While LC–MS shows slightly better sensitivity for some FFAs in negative ESI mode, our SFC-MS method provides comparable sensitivity for most FFAs, particularly medium-chain and long-chain species (C12–C26), and sufficient sensitivity for short-chain FFAs (C4–C8) with minimal ion suppression, while also offering superior separation efficiency, especially for structurally similar FFAs like isomers and unsaturated fatty acids, without requiring extended gradient times or derivatization steps often needed in LC–MS [18, 23, 39].

Absolute quantification was achieved using a stable isotope strategy, with calibration curves based on signature transitions of corresponding deuterated IS, including C17:0 as a conventional IS. The use of deuterium-labeled IS is common for quantitative analysis using techniques like LC–MS/MS or SFC-MS/MS because they behave similarly to the analyte [18, 30]. However, if the retention (or elution) time of the deuterated IS slightly differs from that of the target analyte, it can lead to differences in the matrix effects, which can compromise the accuracy of standardization and quantitation. In fact, C17:0 may not have the exact retention time as shorter- or longer-chain FFAs (e.g., C4 or C26). If matrix effects are time-dependent, the IS might not perfectly correct for all FFAs. Validation studies, including matrix effect assessments and recovery tests, confirmed that C17:0 provides reliable quantitative correction for a wide range of FFAs (C4–C26). Our SIR findings are consistent with previously reported values, confirming the reliability of the method [38].

Using SFC as a primary technique for FFA identification and quantification is relatively new in comparison to other techniques, such as colorimetric, electrochemical, spectroscopic (FT-IR, Raman, nuclear magnetic resonance or NMR), and even with its chromatographic counterparts, GC and LC [19, 20]. SFC was applied for FFA separation across various chain lengths, coupled with different detectors and also with MS [20, 31–33]. For instance, one study separated FFAs from C6 to C26 using an ODS silica-gel column with sCO₂ as the mobile phase, achieving effective separation of eleven (11) even-numbered FFAs under pressure-programmed conditions within 30 min [40]. Another study separated FFAs from C8 to C24 without derivatization in 28 min, using diverse stationary phases and sCO₂, demonstrating the adaptability of SFC for different FFAs chain lengths [35]. A recent study involving FFAs without derivatization was the separation of eight (8) unsaturated FFAs using an SFC-MS method coupled with an online Paternò-Büchi reaction in 10 min [34]. There is no current method in SFC-MS analyzing underivatized FFAs spanning from short-chain to long-chain that is efficient and sensitive enough to cover different sample types. Our method uniquely spans the 31 FFA species from C4 to C26 in a single SFC setup without derivatization and minimal sample preparation within an analytical run of 6 min, distinguishing it from previous works offering efficient, comprehensive analysis.

Method optimization involved selecting suitable stationary phases, starting with generic screening using methanol. The chemistry of the stationary phase proved to be the most critical factor influencing FFA retention [41]. Despite well-characterized stationary phases, selecting the optimal phase remains challenging due to the diverse properties of FFAs. Based on a comprehensive literature review, we selected the 1-AA and HSS C18 columns (Table S3) for their potential to separate FFAs from C4 to C26 [34]. These stationary phases were assessed for their ability to separate a broad range of FFAs, a key aspect of method development. FFA elution pattern in SFC is influenced by interactions with the stationary phase, along with the carbon chain length, degree of unsaturation, and spatial orientation [3, 34, 35]. The 1-AA stationary phase is less effective for FFA separation compared to HSS C18 due to differences in column chemistry (Table S3). FFAs, being largely nonpolar, require strong hydrophobic interactions for effective retention and separation. The polar amine group of the 1-AA column does not provide the necessary hydrophobic environment, whereas the HSS C18 column, with its nonpolar C18 alkyl chains, facilitates strong van der Waals interactions [34]. This setup allows effective separation of FFAs across C4 to C26, following reverse-phase-like chromatography principles in SFC. This makes the HSS C18 column more suitable for comprehensive FFAs analysis, as it offers superior retention, resolution, and selectivity. Under optimized chromatographic conditions, the HSS C18 SB column achieved near-baseline separation for both saturated and unsaturated FFAs, exhibiting symmetrical peaks and relatively short retention times, but unique [M − H]⁻ ions of each FFA from the SIM mode in the MS will distinguish the species, hence performing accurate quantification. Saturated FFAs eluted in order of increasing alkyl chain length, with shorter-chain FFAs eluting earlier, likely due to weaker interactions with the C18-bonded phase. Interestingly, unsaturated FFAs showed longer retention times as the number of double bonds decreased, suggesting that FFAs with fewer double bonds interact more strongly with the HSS C18 SB phase, potentially due to reduced conformational flexibility and increased surface contact.

The selection of organic co-solvent is crucial for effective separation in SFC. Adding formic acid to methanol as a modifier provided better peak shapes compared to ammonium formate. Formic acid creates a mildly acidic environment that stabilizes the carboxylic acid group in FFAs. The acidic pH environment helps retain the (H^+^) of on the carboxylic acid group, reducing unwanted interactions with the stationary phase. This stabilization leads to more consistent elution and sharper peak shapes. On the other hand, many stationary phases, especially silica-based ones, have residual silanol groups that can interact with polar compounds, causing peak tailing or broadening. The addition of formic acid reduces these interactions by neutralizing the silanol groups, which prevents unwanted retention and peak distortion, resulting in improved peak shapes. In fact, formic acid has better polarity matching with methanol in SFC compared to ammonium formate. Formic acid acts as a reliable proton donor, which can help maintain FFAs in a protonated, stable form throughout separation [42]. Additionally, the makeup solvent composition is essential for the ionization efficiency of FFAs in MS. Methanol with ammonium hydroxide as an additive for the makeup solvent resulted in higher signal intensities for FFAs compared to ammonium fluoride. Ammonium hydroxide creates a more basic environment in the makeup solvent, which enhances the deprotonation of FFAs. This basic environment favors the formation of [M − H]⁻ ions in negative electrospray ionization (ESI^−^), leading to more efficient ionization and thus higher signal intensities. Ammonium hydroxide is less likely to produce complex adducts with FFAs compared to ammonium fluoride. Ammonium fluoride can introduce fluoride ions (F⁻), which may form unwanted adducts or ion clusters, leading to reduced ionization efficiency and signal suppression. Overall, the use of ammonium hydroxide as an additive facilitates more effective deprotonation and cleaner ionization, leading to improved sensitivity and higher signal intensities for FFAs in ESI^−^ mode.

The elution profile of FFAs is influenced not only by their interaction with the stationary phase but also by their logP (partition coefficient) values (Fig. 1). logP reflects the relative affinity of a compound for polar (water) versus non-polar (octanol) environments. SCFFAs, with lower logP values, favoring interaction with the mobile phase over the stationary phase, which results to shorter retention times (Table 1). For FFAs of similar chain length, logP is also affected by the degree of unsaturation. As the number of double bonds increases, logP decreases, leading to shorter retention times for unsaturated FFAs with the same chain length.

To extract FFAs from pharmaceutical-grade egg yolk powders, we optimized a simple liquid–liquid extraction based on Dole’s method [43]. The Dole method is designed specifically to extract FFAs without requiring derivatization. This makes it ideal for direct FFA analysis in their native form, preserving the integrity of FFAs and simplifying the analysis. By combining an aqueous phase with an organic phase like heptane, the Dole method efficiently separates FFAs from other lipid components and impurities (salts). The acid hydrolysis step helps release FFAs from esterified forms (such as triglycerides or phospholipids), allowing for better phase separation and FFAs recovery.

### SFC-MS analytical method validation

The analytical compliance of the developed SFC-MS method was tested according to ICH Q2(R2) guidelines: lower limit of quantification (LLOQ), linearity, accuracy, precision, recovery, matrix effect, and stability. Performing analytical validation ensures method applicability, since there is no currently published method that allows rapid quantification of FFAs from C4 to C26 in SFC-MS without chemical derivatization. Matrix-matched and solvent calibration curves were constructed to assess the influence of matrix effects in FFA response at standard concentrations (Fig. S11). The LLOQ for each FFA standard was determined through individual injections in the SFC-MS system, ensuring a signal-to-noise (S/N) ratio greater than 10. Most FFAs were detected as low as 50 ng/mL, except C4:0, C6:0, and C8:0. The LLOQ for SCFFAs was obtained at 1000 ng/mL. Overall, this method showed greater sensitivity than similar protocols on FFA analysis using SFC, which had detection limits of either 0.1 µg/mL [3], 0.1 mg/mL [44], or 1000 ppm [45], making it a hundred to a thousand times more sensitive. These LLOQ values were used as the first calibration points of the standard curves. Two separate calibration ranges were established for solvent and matrix-matched curves for analytical method validation due to the higher LLOQs of SCFFAs.

All FFAs showed strong linearity (*R*^2^ ≥ 0.9910) across the working range for both solvent and matrix-matched conditions. Most FFAs obtained similar slopes, indicating that the charcoal adsorption method was effective to eliminate most of the FFAs present in the tested egg yolk powder that produces significant matrix effects (Fig. S11). However, C18:0 and C18:1 showed different linearities in the calibration models compared to the other FFAs. The observed difference in slopes was due to their endogenous nature in the used matrix, in contrast to other FFAs [17, 28]. Reducing matrix effects for these FFAs is challenging within LLOQ due to the decreased analytical sensitivity at this concentration level. To compensate, the addition of quality control (QC) samples at five different concentration levels tests method reliability, monitoring its accuracy and precision. In this study, the lowest QC sample (QC 1) was set at the limit of detection (LOD, S/N > 3), as the LLOQ was determined from the lowest calibration point (Table S4). This approach helps minimize false positives in real sample analysis. Conversely, the highest QC level (QC 5) was set significantly above the last calibration point. Testing accuracy and precision at lower and higher concentration extremes of the working range provide insights in FFA behavior in matrix in addition to checking the robustness of the developed SFC-MS method (Table S4). Comparing these results to the existing LC- and GC–MS techniques for FFA quantification that operates mostly in the 1 to 200 µg/mL range [17], which can also be more sensitive to 100 to 1000 ng/mL [23], or even detected as low as 10 nM especially when a twin-derivatization technique is applied [18], our method is considered equally to more sensitive to these standard methods. However, the lack of derivatization step in our method development makes our analysis more efficient and less prone to analyte losses and does not compensate sensitivity and accuracy in quantification, as observed from our figures of merit.

The accuracy of the SCFFAs was compliant from QC 2 to QC 5 (bias at ± 15). For C4:0 at QC 1, the accuracy was acceptable at 10.6% (bias at ± 20%, ≤ LLOQ). However, C6:0 (38.5%) and C8:0 (23.1%) did not meet validation requirements at this level that can be correlated with the use of C4:0-*d*_7_ as IS for these analytes, which explains the acceptable accuracy for C4:0 but not for C6:0 and C8:0 at QC 1. It is recommended for future studies to employ individual internal standards for SCFFAs, since the difference in chain structure in these FFA species has a huge impact in their chemical properties, as compared to MC- and LCFFAs. Intraday precision and interday precision for the SCFFAs were non-compliant at QC 1 (%CV at ± 20%, ≤ LLOQ), but compliant at higher QC levels (%CV at ± 15%), indicating that measurement reliability is best within the calibration range (1000–12,000 ng/mL), as shown in Fig. 2. The FFAs from C10 to C26 were also compliant for accuracy and precision at QC levels 2 to 5. Huge measurement uncertainties were found in QC 1 for some FFAs, particularly C18:0, which had a bias of 70% and %CVs for intraday and interday precision of 47.4% and 71.0%, respectively. At QC 2, the accuracy of C18:0 was acceptable, but variability remained high, with %CVs of 44.4% and 56.7% for intraday and interday precision (Fig. 3). This variability is linked to the endogenous nature of C18:0, which decreases measurement reliability at concentrations within LLOQ. However, since the samples are expected to contain higher levels of C18:0, the developed method is suitable enough for its analysis along with the other 30 FFAs.

Recovery measurements through spiking FFA standards aim to refine the sample extraction procedure for pharmaceutical-grade egg yolk powders (Fig. 4). It was expected that C18:0 would show the highest recovery, as some residual C18:0 might remain in the matrix even after the charcoal adsorption procedure. However, C14:1 and C15:0 showed the highest recovery values at 128% and 124%, respectively. These FFAs are not typically found in high amounts; C14:1 present in animals, though it is generally found in low concentrations, and C15:0 is an uncommon odd-numbered fatty acid present in trace amounts in ruminant animal fats and dairy products [46, 47]. Systematic carryover tests indicated that C14:1 and C15:0 produced larger carryover peaks than C18:0. The difference in the carbon chain length of the IS for C15:0, C10:0-*d*_19_, may have influenced the wider recovery range. For the real sample analysis, additional blank injections were performed after a 4-min cleanup following the analytical run to compensate for these systematic carryovers.

Stability studies are essential for evaluating long-term retention and degradation. Given that PLEs are multicomponent substances including FFAs, understanding their optimal storage conditions is crucial. Adhering to these conditions preserves analyte integrity and ensures the sensitivity of our SFC-MS method. The first freeze–thaw cycle showed higher variability than the second and third cycles, likely due to the initial temperature shock experienced by the analyte, which decreases over time (Fig. 5). QC 1 had higher %CV compared to other QC levels, with %CV decreasing as FFA concentrations increase. This implies that FFAs in low concentrations are more matrix and temperature sensitive. Among FFA species, greater variations were found in SCFFAs since they are volatile, sensitive to phase transitions during the freeze–thaw cycles, and susceptible to thermal degradation, making their quantification more affected by temperature than most FFAs.

Increased measurement variability was observed in the autosampler stability study for FFAs (Fig. 6). QC 1 had the highest variability, to which SCFFAs and MCFFAs showed greater variability after 24 h, as opposed to LCFFAs with most variations after 72 h. This pattern suggests that the stability of SCFFAs and MCFFAs aligns with the freeze–thaw findings, where initial temperature changes caused more variability, and less variations were seen over time. In contrast, the significant increase in measurement variability for LCFFAs suggests chemical degradation, especially the unsaturated ones since they can undergo oxidation.

### FFA analysis in pharmaceutical-grade egg yolk powders using SFC-MS

**Fig. 7:**
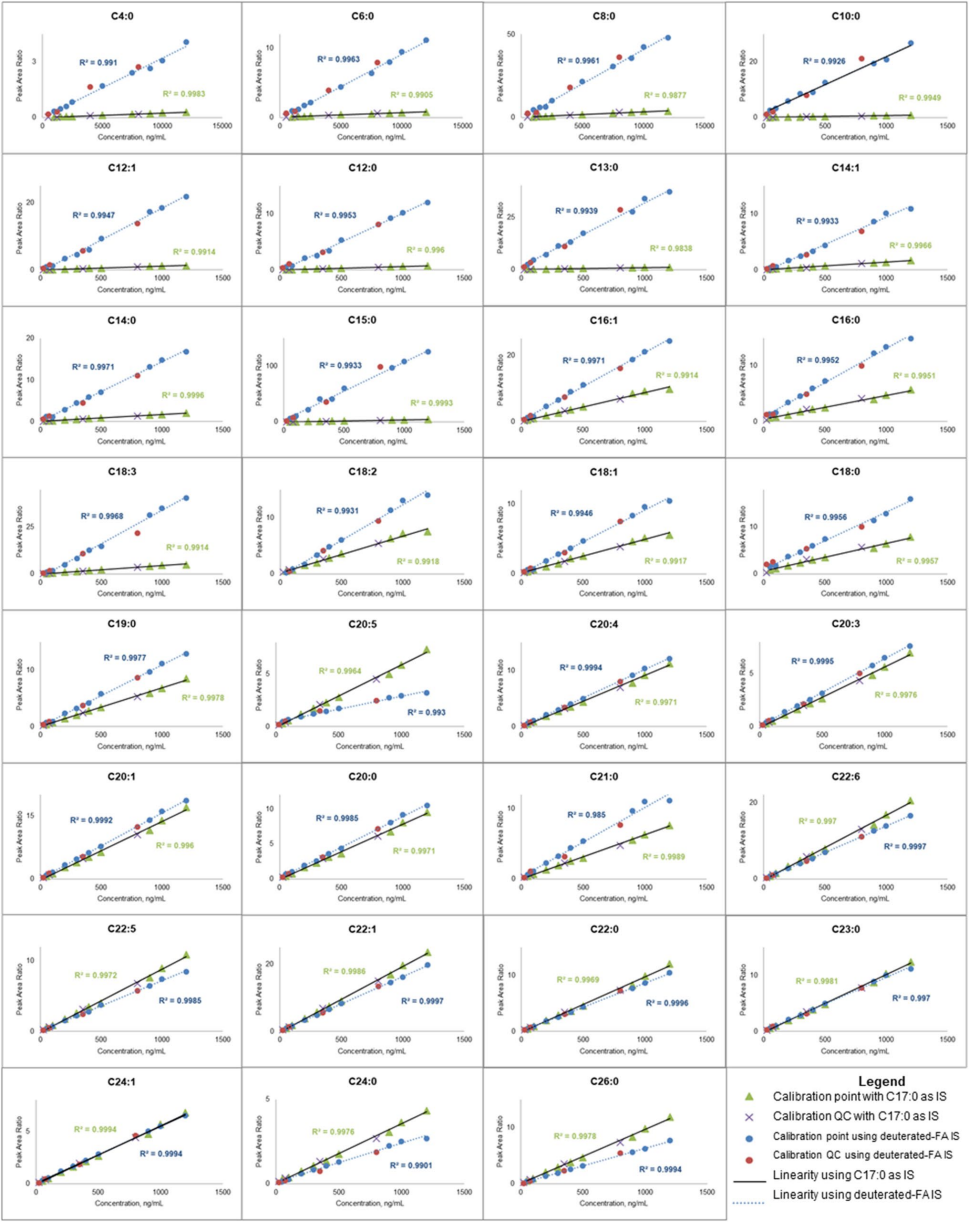
Construction of matrix-matched calibration curves using deuterated FFAs and C17:0 as internal standards in the validated SFC-MS method

The validated SFC-MS method was applied for identification and quantification of FFAs present in pharmaceutical-grade egg yolk powders using deuterated FFAs IS, taking advantage of their similar chemical behavior. Additionally, heptadecanoic or margaric acid (C17:0), a non-endogenous, odd-numbered fatty acid served as an alternative IS to explore a more practical quantification approach. Both IS models produced satisfactory matrix-matched calibration curves (*R*^2^ ≥ 0.9901), but the slopes for quantifying individual FFAs varied significantly (Fig. 7). Slopes using C17:0 were generally less steep than those with the deuterated IS, with the calibration curves converging as the carbon chain length increased. For instance, the slopes for C18:0 were most similar between the two IS methods, while unsaturated FFAs (C18:1, C18:2, C18:3) showed greater divergence. This suggests that C17:0 is more suitable for quantifying C18:0 compared to its unsaturated counterparts due to their structural similarity in absence of a deuterated IS. Notably, C17:0 yielded higher slopes than the deuterated IS for long-chain FFAs (C21:0 and above), indicating that the C17:0 IS may overestimate LCFFA content compared to its deuterated counterpart.

**Fig. 8:**
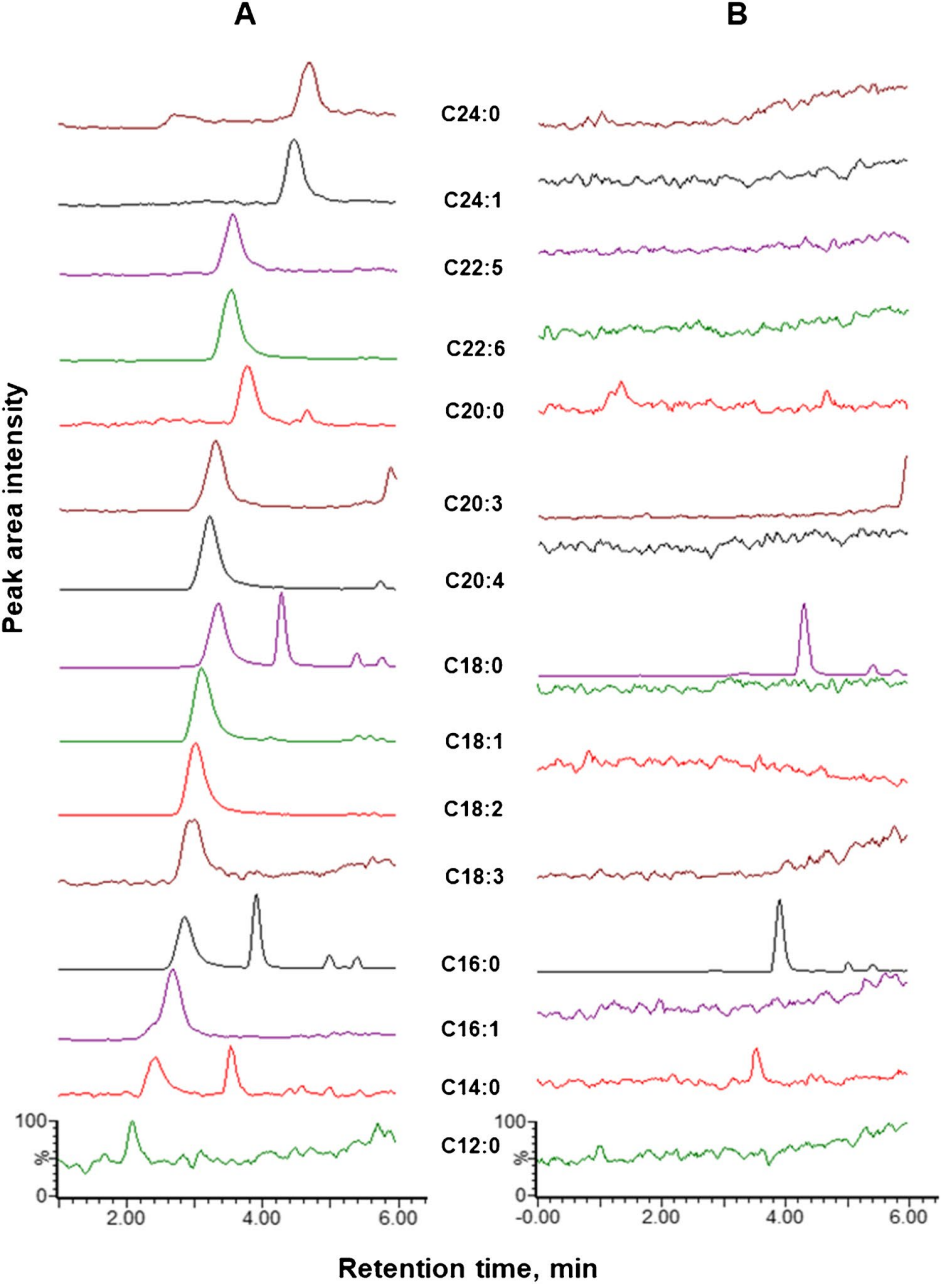
Layered selected ion recording (SIR) chromatograms of 15 detected FFAs present in a **A** pharmaceutical-grade egg yolk sample using the validated LC–MS method together with the **B** blank matrix

The validated SFC-MS method detected fifteen (15) FFAs were detected in pharmaceutical-grade egg yolk powders (Fig. 8). Blank matrix readings were used as references to compare SIR chromatograms of detected FFAs. Additional peaks observed for some FFAs, aside from the distinct peak from the validated method originated from the blank matrix, confirming no sample carryover. This underscores the accuracy and reliability of the method, especially when using a matrix-matched approach, enhancing confidence in analytical quantification and supporting its application in pharmaceutical quality assurance (QA) and quality control (QC).

The most abundant FFAs found in the samples were C16:0, C18:1, and C18:0 (Table 2), along with unsaturated FFAs such as arachidonic acid (C20:4) and docosahexaenoic acid (DHA, C22:6). Saturated FFAs were present in significant amounts, while SCFFAs were not detected. These findings are consistent with previous studies on FFA quantification in egg-based pharmaceutical samples [17, 26, 28, 48, 49]. Although SCFFAs were absent, including them in our SFC-MS method broadens the method’s applicability to a wider range of samples and matrices.

**Table 2:** Analytical values obtained from two quantification approaches applied for 31 FFAs in 3 pharmaceutical-grade egg yolk powders (*n* = 2) using the validated SFC-MS method

Compound		IS: C17:0			IS: *d*-labelled FFAs (13 in total)		
		Concentration, mg FFA/kg sample			Concentration, mg FFA/kg sample		
		S1	S2	S3	S1	S2	S3
1	C4:0	ND	ND	ND	ND	ND	ND
2	C6:0	ND	ND	ND	< LLOQ	< LLOQ	< LLOQ
3	C8:0	ND	ND	ND	ND	ND	ND
4	C10:0	ND	ND	ND	< LLOQ	< LLOQ	< LLOQ
5	C12:1	ND	ND	ND	ND	ND	ND
6	C12:0	14.7	15.6	12.9	29.1	41.7	31.0
7	C13:0	< LLOQ	< LLOQ	< LLOQ	< LLOQ	< LLOQ	< LLOQ
8	C14:1	ND	ND	ND	ND	ND	ND
9	C14:0	23.7	24.3	23.8	22.1	20.0	18.7
10	C15:0	< LLOQ	< LLOQ	< LLOQ	ND	ND	ND
11	C16:1	18.7	20.7	20.4	114.0	119.0	113.2
12	C16:0	5860.0	8562.9	5969.9	66,712.9	94,207.8	54,595.3
13	C18:3	7.9	10.5	10.1	14.0	18.3	19.1
14	C18:2	215.5	273.0	277.0	193.9	206.5	210.3
15	C18:1	2622.8	2930.3	2829.2	27,863.2	33,430.2	32,003.0
16	C18:0	9378.5	12,407.3	8676.4	111,724.2	111,184.1	106,836.3
17	C19:0	< LLOQ	< LLOQ	< LLOQ	ND	ND	ND
18	C20:5	< LLOQ	< LLOQ	< LLOQ	ND	ND	ND
19	C20:4	82.1	111.9	115.6	98.3	150.2	163.1
20	C20:3	19.0	24.8	25.6	20.1	30.4	33.3
21	C20:1	< LLOQ	< LLOQ	< LLOQ	< LLOQ	< LLOQ	< LLOQ
22	C20:0	20.9	20.4	19.0	24.0	26.4	26.0
23	C21:0	ND	ND	ND	ND	ND	ND
24	C22:6	32.1	42.8	43.4	39.7	56.6	59.3
25	C22:5	22.0	29.8	29.4	26.9	39.1	39.9
26	C22:1	< LLOQ	< LLOQ	< LLOQ	ND	ND	ND
27	C22:0	< LLOQ	< LLOQ	< LLOQ	< LLOQ	< LLOQ	< LLOQ
28	C23:0	< LLOQ	< LLOQ	< LLOQ	ND	ND	ND
29	C24:1	23.4	21.6	19.4	23.7	23.3	21.6
30	C24:0	43.6	34.8	31.9	64.2	54.8	52.2
31	C26:0	ND	ND	ND	ND	ND	ND

*ND*, not detected; < *LLOQ*, less than the lower limit of quantification

The observed differences in FFA quantification between the two IS approaches highlight the importance of selecting an appropriate IS. Although C17:0 provides satisfactory analytical performance, using a more chemically relevant IS, such as a deuterated standard, may yield more accurate results. We recommend first validating the method with a deuterated IS, followed by a comparison with C17:0 to assess quantification differences through response factors. This study demonstrates the robustness of the validated SFC-MS method, which remains reliable despite the use of different quantification approaches.

## Conclusions

A novel SFC-MS method was successfully developed to analyze 31 FFAs using 13 deuterated IS without the need of chemical derivatization within an analytical run time of 6 min. This method effectively quantifies FFAs across a wide range of chain lengths, from short- to long-chain FFAs (C4 to C26), making it applicable for diverse uses in pharmaceutical quality control, clinical research, and nutritional analysis. Matrix-matched analytical validation was conducted, and the method was successfully applied to pharmaceutical-grade egg yolk samples. Two quantification methods were explored: one using the deuterated FFA standards and the other using C17:0, a common IS, enhancing the method’s accessibility for different sample types, including biological, clinical, and pharmaceutical samples. The method allows for FFA analysis across different carbon chain lengths and unsaturation levels without requiring extensive pre-treatment, allowing direct analysis without derivatization. Our method has expanded analytical range within FFA species providing enhanced separation efficiency and improved practicality and sustainability. To our knowledge, no published studies are available for direct comparison with our results, underscoring the novelty of this method and its contributions to FFA analysis in pharmaceutical-grade samples.

## Supplementary Information

Below is the link to the electronic supplementary material.

Supplementary file1 (DOCX 2.26 MB)
