# Supplementary material for: Comprehensive quantification of C4 to C26 free fatty acids using a supercritical fluid chromatography-mass spectrometry method in pharmaceutical-grade egg yolk powders intended for total parenteral nutrition use
Source: Anal Bioanal Chem. 2025 Jan 23;417(8):1461–78. doi: 10.1007/s00216-025-05732-3 (PMC11876226; doi:10.1007/s00216-025-05732-3)
Supplement: Supplementary file 1 — Supplementary file1 (DOCX 2.26 MB) [file 216_2025_5732_MOESM1_ESM.docx]

**Supplementary Information**

provided for

**Comprehensive quantification of C4 to C26 free fatty acids using a supercritical fluid chromatography-mass spectrometry method in pharmaceutical-grade egg yolk powders intended for total parenteral nutrition use**

Mark Dennis Chico Retrato, Anh Vu Nguyen, S. J. Kumari A. Ubhayasekera*, and Jonas Bergquist*

Department of Chemistry – Biomedical Center, Analytical Chemistry and Neurochemistry, Uppsala University, Uppsala, Sweden

***Supplementary Tables***

**Table S1** Structural information of the FFA standards used as analytes for the SFC-MS method

**Table S2** Structural information of the deuterated FFAs and C17:0 used as internal standards for the SFC-MS method

**Table S3** Specifications of the SFC columns used for stationary phase screening

**Table S4** Analytical figures of merit for 31 FFA standards in the developed SFC-MS method using 5 QC concentration levels

***Supplementary Figures***

**Fig. S1** Gradient and flow rate optimization for FFA analysis in SFC-MS using (A) 0.6, (B) 0.8, (C) 1.0, (D) 1.2, (E) 1.5 mL/min

**Fig. S2** Effects of makeup solvent modification in SFC-MS using (A) NH_4_OH and (B) NH_4_F in saturated FFA standards

**Fig. S3** Effects of makeup solvent modification in SFC-MS using (A) NH_4_OH and (B) NH_4_F in unsaturated FFA standards

**Fig. S4** SFC-MS overlaid chromatograms of the 31 FFA standards using the Torus 1-AA column

**Fig. S5** SFC-MS detection of SCFFAs (C4:0, C6:0, and C8:0) in the HSS C18 column

**Fig. S6** Selected ion recording (SIR) chromatogram of heptadecanoic acid, C17:0 as an internal standard for the quantification of pharmaceutical-grade egg yolk powders

**Fig. S7** Selected ion recording (SIR) chromatograms of 13 deuterated FFAs used as internal standards for the quantification of pharmaceutical-grade egg yolk powders

**Fig. S8** SFC-MS overlaid chromatograms of 31 FFA standards using the HSS C18 column

**Fig. S9** SFC-MS overlaid chromatograms of 14 deuterated FFA internal standards using the HSS C18 column

**Fig. S10** Evaluation of systematic carryovers in SFC-MS method in analyzing 31 FFAs

**Fig. S11** Construction of the solvent and matrix-matched calibration curves for 31 FFA standards using the deuterated FFA internal standards

***Supplementary Tables***

**Table S1** Structural information of the FFA standards used as analytes for the SFC-MS method

|  | **Name** | **Notation** | **Formula** | **MW** | **Structure** |
| --- | --- | --- | --- | --- | --- |
| 1 | Butyric acid | C4:0 | C_4_H_8_O_2_ | 88.05 | 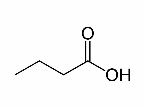 |
| 2 | Caproic acid | C6:0 | C_6_H_12_O_2_ | 116.08 | 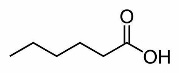 |
| 3 | Caprylic acid | C8:0 | C_8_H_16_O_2_ | 144.12 | 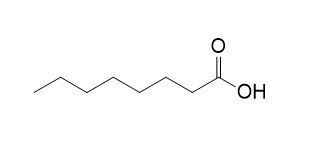 |
| 4 | Capric acid | C10:0 | C_10_H_20_O_2_ | 172.15 | 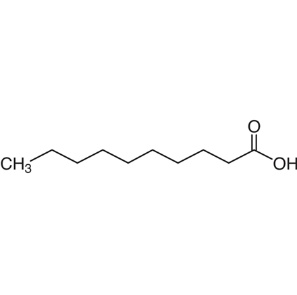 |
| 5 | Lauroleic acid | C12:1 | C_12_H_22_O_2_ | 198.16 | 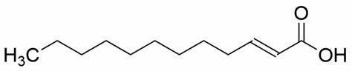 |
| 6 | Lauric acid | C12:0 | C_12_H_24_O_2_ | 200.18 | 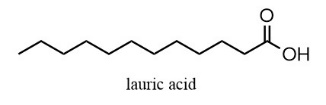 |
| 7 | Tridecylic acid | C13:0 | C_13_H_26_O_2_ | 214.19 | 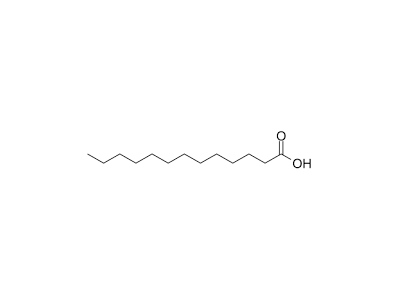 |
| 8 | Myristoleic acid | C14:1 | C_14_H_26_O_2_ | 226.19 | 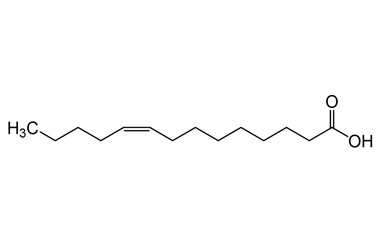 |
| 9 | Myristic acid | C14:0 | C_14_H_28_O_2_ | 228.21 | 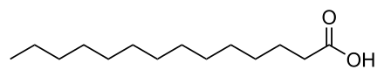 |
| 10 | Pentadecylic acid | C15:0 | C_15_H_30_O_2_ | 242.22 | 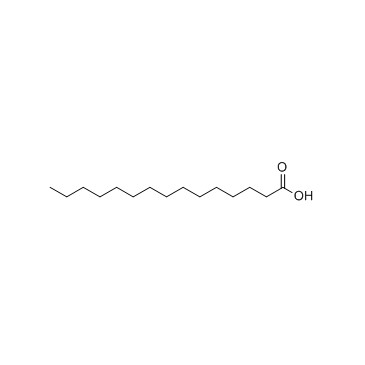 |
| 11 | Palmitoleic acid | C16:1 | C_16_H_30_O_2_ | 254.22 | 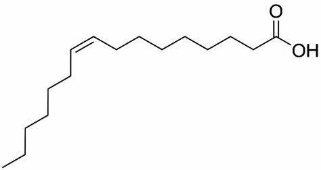 |
| 12 | Palmitic acid | C16:0 | C_16_H_32_O_2_ | 256.24 | 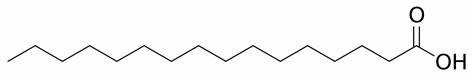 |
| 13 | α-Linolenic acid | C18:3 | C_18_H_30_O_2_ | 278.22 | 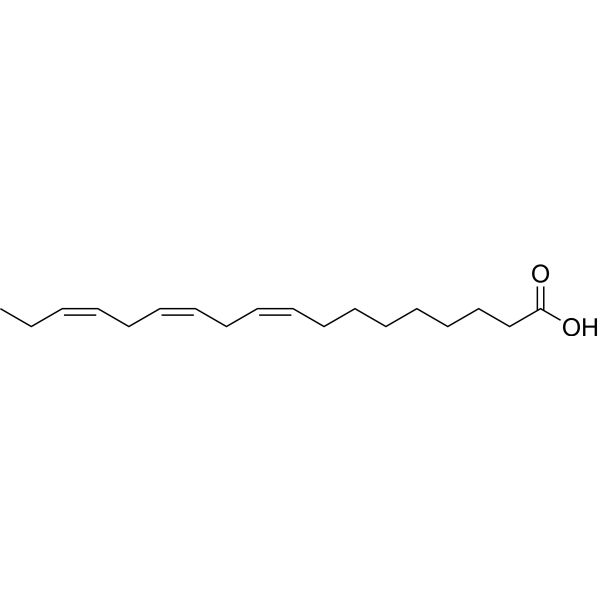 |
| 14 | Linoleic acid | C18:2 | C_18_H_32_O_2_ | 280.24 | 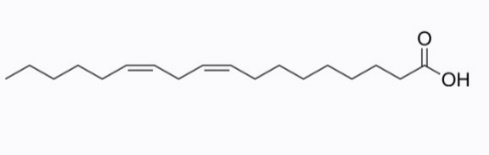 |
| 15 | Oleic acid | C18:1 | C_18_H_34_O_2_ | 282.26 | 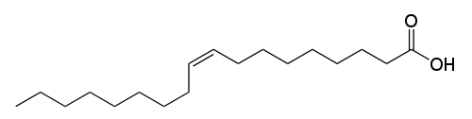 |
| 16 | Stearic acid | C18:0 | C_18_H_36_O_2_ | 284.27 | 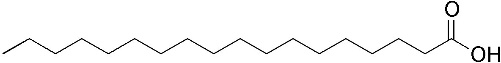 |
| 17 | Nonadecylic acid | C19:0 | C_19_H_38_O_2_ | 298.29 | 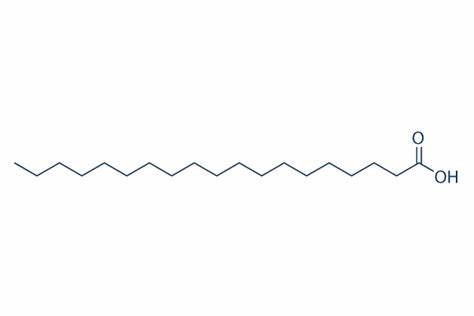 |
| 18 | Eicosapentanoic acid | C20:5 | C_20_H_30_O_2_ | 302.22 | 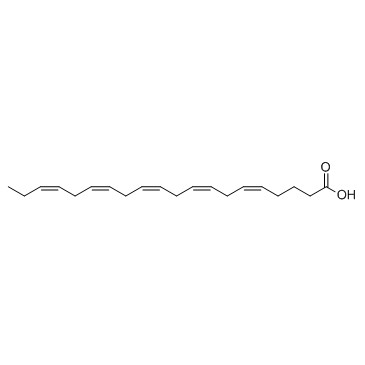 |
| 19 | Arachidonic acid | C20:4 | C_20_H_32_O_2_ | 304.24 | 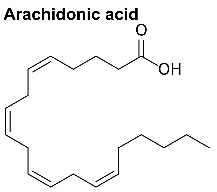 |
| 20 | Eicosatrienoic acid | C20:3 | C_20_H_34_O_2_ | 306.26 | 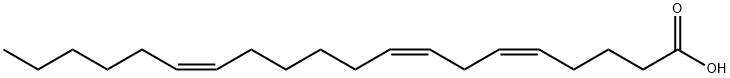 |
| 21 | Eicosenoic acid | C20:1 | C_20_H_38_O_2_ | 310.29 | 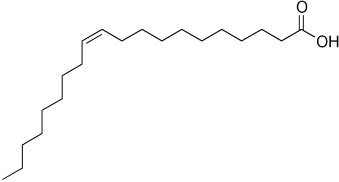 |
| 22 | Arachidic acid | C20:0 | C_20_H_40_O_2_ | 312.30 | 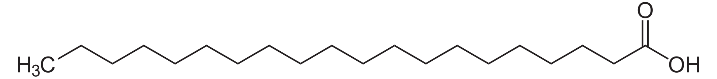 |
| 23 | Heneicosylic acid | C21:0 | C_21_H_42_O_2_ | 326.32 | 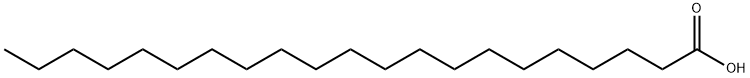 |
| 24 | Docosahexaenoic acid | C22:6 | C_22_H_32_O_2_ | 328.24 | 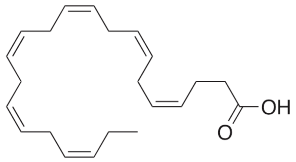 |
| 25 | Docosapentaenoic acid | C22:5 | C_22_H_34_O_2_ | 330.26 | 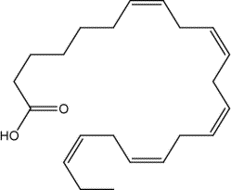 |
| 26 | Erucic acid | C22:1 | C_22_H_42_O_2_ | 338.32 | 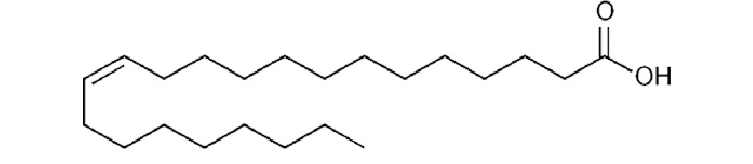 |
| 27 | Behenic acid | C22:0 | C_22_H_44_O_2_ | 340.33 | 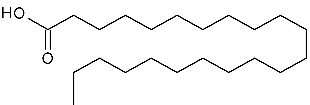 |
| 28 | Tricosylic acid | C23:0 | C_23_H_46_O_2_ | 354.35 | 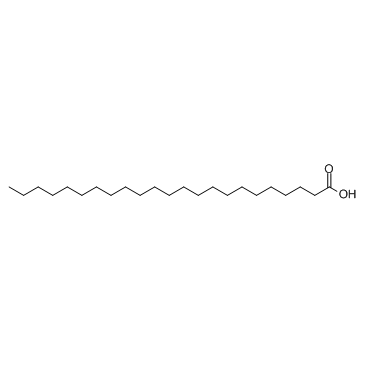 |
| 29 | Nervonic acid | C24:1 | C_24_H_46_O_2_ | 366.35 | 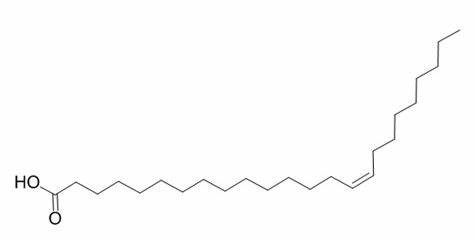 |
| 30 | Lignoceric acid | C24:0 | C_24_H_48_O_2_ | 368.37 | 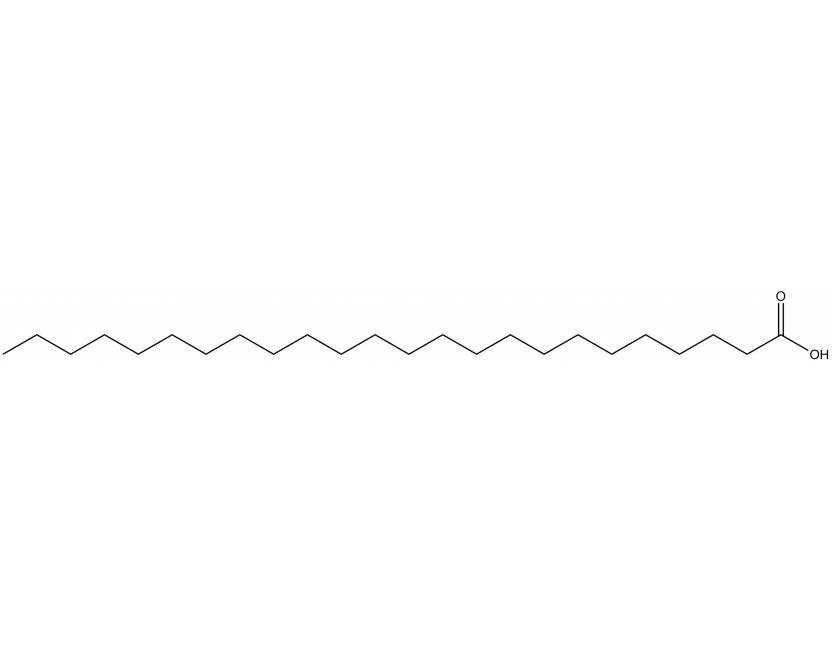 |
| 31 | Cerotic acid | C26:0 | C_26_H_52_O_2_ | 396.40 | 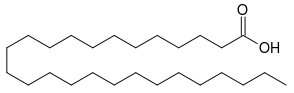 |

**Table S2** Structural information of the deuterated FFAs and C17:0 used as internal standards for the SFC-MS method

|  | **Notation** | **Formula** | **MW** | **Structure** | **Monitored Analyte** |
| --- | --- | --- | --- | --- | --- |
| 1 | C4:0-*d*7 | C_4_HD_7_O_2_ | 95.10 | 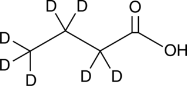 | C4:0, C6:0, and C8:0 |
| 2 | C10:0-*d*19 | C_10_HD_19_O_2_ | 191.38 | 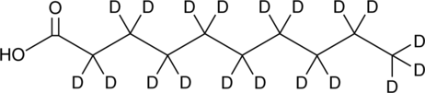 | C10:0, C13:0, and C15:0 |
| 3 | C12:0-*d*23 | C_12_HD_23_O_2_ | 223.50 | 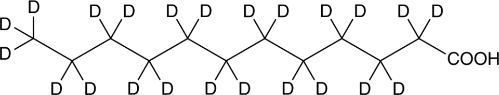 | C12:0 and C12:1 |
| 4 | C14:0-*d*27 | C_14_HD_27_O_2_ | 255.52 | 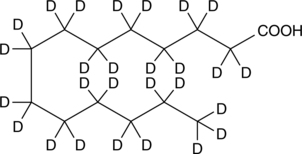 | C14:0 and C14:1 |
| 5 | C16:1-*d*14 | C_16_H_16_D_14_O_2_ | 268.44 | 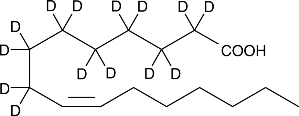 | C16:1 |
| 6 | C16:0-*d*31 | C_16_HD_31_O_2_ | 287.60 | 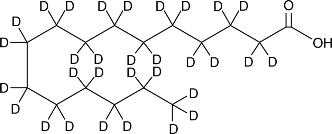 | C16:0 |
| 7 | C18:0-*d*35 | C_18_HD_35_O_2_ | 319.68 | 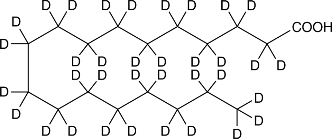 | C18:0 and C19:0 |
| 8 | C18:1-*d*17 | C_18_H_17_D_17_O_2_ | 299.57 | 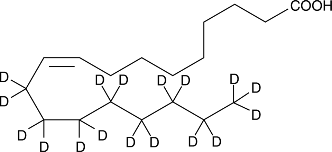 | C18:1 |
| 9 | C18:2-*d*4 | C_18_H_28_D_4_O_2_ | 284.50 | 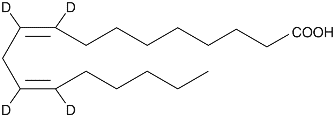 | C18:2 |
| 10 | C18:3-*d*5 | C_18_H_25_D_5_O_2_ | 283.51 | 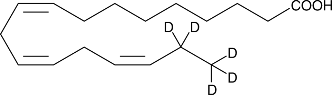 | C18:3 |
| 11 | C20:4-*d*11 | C_20_H_21_D_11_O_2_ | 315.46 | 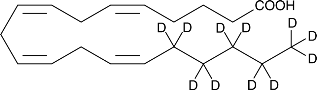 | C20:0, C20:1, C20:2, C20:3, and C21:0 |
| 12 | C20:5-*d*5 | C_20_H_25_D_5_O_2_ | 307.54 | 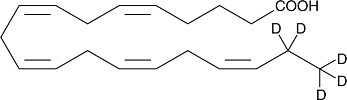 | C20:5 |
| 13 | C22:6-*d*5 | C_22_H_27_D_5_O_2_ | 333.52 | 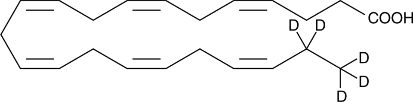 | C22:0, C22:1, C22:5, C22:6, C23:0, C24:0, C24:1, C26:0 |
| 14 | C17:0 | C_17_H_34_O_2_ | 270.26 | 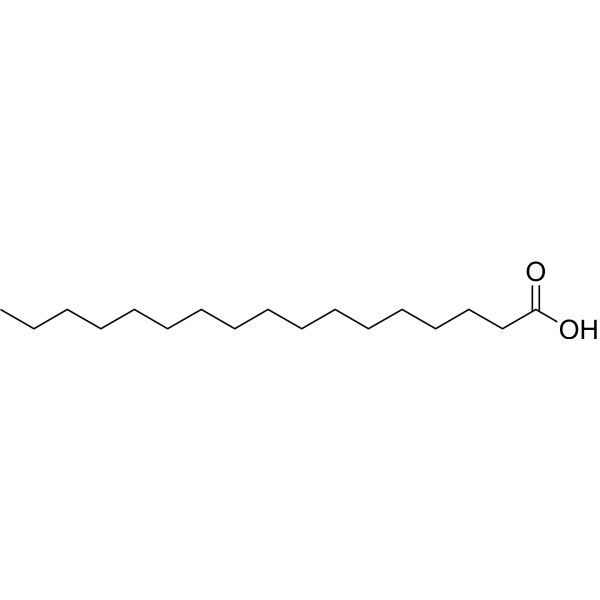 | All of the 31 FA standards |

**Table S3** Specifications of the SFC columns used for stationary phase screening

| Column | Pore size, Å | Particle size, µm | Column dimensions, mm | Structure |
| --- | --- | --- | --- | --- |
| Torus 1-AA | 130 | 1.7 | 2.1 x 100 | 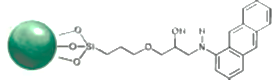 |
| Acquity UPLC HSS C18 SB | 100 | 1.8 | 3.0 x 100 | 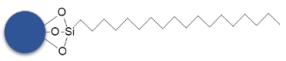 |

**Table S4** Analytical figures of merit for 31 FFA standards in the developed SFC-MS method using 5 QC concentration levels

|  |  |  | **Q1** | | | **Q2** | | | **Q3** | | | **Q4** | | | **Q5** | | | Recovery, % |
| --- | --- | --- | --- | --- | --- | --- | --- | --- | --- | --- | --- | --- | --- | --- | --- | --- | --- | --- |
|  | Linear range, ng/mL | LLOQ, ng/mL | Accuracy (%Bias) | Precision | | Accuracy (%Bias) | Precision | | Accuracy (%Bias) | Precision | | Accuracy (%Bias) | Precision | | Accuracy (%Bias) | Precision | |  |
|  |  |  |  | Intraday  (%CV) | Interday (%CV) |  | Intraday  (%CV) | Interday (%CV) |  | Intraday  (%CV) | Interday (%CV) |  | Intraday  (%CV) | Interday (%CV) |  | Intraday  (%CV) | Interday (%CV) |  |
| **C4:0** | 1000-12000 | 1000 | 10.6% | 29.2% | 21.5% | -5.9% | 16.7% | 16.4% | -1.4% | 9.6% | 10.1% | 0.4% | 7.8% | 7.8% | 3.4% | 14.9% | 11.9% | 98 |
| **C6:0** | 1000-12000 | 1000 | 38.5% | 31.2% | 34.9% | 0.9% | 13.3% | 11.7% | -0.3% | 10.3% | 10.1% | -3.4% | 8.1% | 10.3% | -1.2% | 9.6% | 10.8% | 101 |
| **C8:0** | 1000-12000 | 1000 | 23.1% | 22.7% | 23.1% | -4.9% | 14.1% | 13.3% | 2.3% | 10.3% | 11.6% | 0.6% | 9.2% | 8.3% | 4.1% | 10.2% | 11.0% | 89 |
| **C10:0** | 50-1200 | 50 | -1.2% | 72.3% | 41.0% | -1.5% | 36.9% | 30.1% | -3.5% | 9.3% | 6.6% | 3.9% | 11.6% | 10.3% | -7.1% | 9.8% | 9.0% | 112 |
| **C12:1** | 50-1200 | 50 | 15.4% | 38.0% | 27.3% | 7.0% | 18.7% | 14.5% | -6.2% | 8.2% | 6.8% | -6.1% | 8.0% | 8.5% | -5.7% | 3.5% | 7.1% | 89 |
| **C12:0** | 50-1200 | 50 | -6.7% | 42.4% | 34.5% | 9.2% | 17.2% | 16.2% | -4.9% | 9.8% | 8.4% | -5.8% | 5.8% | 7.3% | -5.3% | 5.8% | 7.4% | 82 |
| **C13:0** | 50-1200 | 50 | -8.5% | 40.4% | 34.8% | -3.2% | 19.5% | 16.5% | -2.2% | 11.5% | 8.3% | 0.3% | 9.8% | 9.2% | -8.7% | 8.1% | 7.3% | 101 |
| **C14:1** | 50-1200 | 50 | 16.1% | 24.8% | 25.0% | 6.9% | 27.9% | 23.8% | 2.5% | 14.7% | 13.0% | 0.5% | 8.9% | 7.6% | -5.1% | 10.6% | 10.3% | 128 |
| **C14:0** | 50-1200 | 50 | 1.5% | 30.3% | 24.8% | 0.4% | 13.5% | 11.3% | -6.3% | 7.5% | 7.6% | -4.5% | 8.9% | 8.0% | -5.6% | 9.1% | 9.1% | 91 |
| **C15:0** | 50-1200 | 50 | 2.7% | 23.1% | 41.0% | -2.1% | 34.8% | 29.5% | -2.5% | 11.2% | 8.2% | 2.2% | 8.5% | 8.2% | -3.9% | 9.8% | 9.1% | 52 |
| **C16:1** | 50-1200 | 50 | 4.2% | 12.9% | 11.3% | 10.5% | 6.9% | 7.0% | -4.3% | 4.2% | 4.2% | -3.5% | 4.5% | 4.1% | -8.7% | 4.8% | 3.9% | 102 |
| **C16:0** | 50-1200 | 50 | 39.4% | 17.5% | 49.2% | -10.3% | 16.6% | 17.6% | -5.7% | 7.6% | 6.6% | -4.8% | 4.7% | 3.6% | -9.3% | 5.7% | 4.9% | 103 |
| **C18:3** | 50-1200 | 50 | 35.7% | 15.5% | 16.9% | 4.7% | 8.6% | 6.4% | -7.2% | 7.7% | 8.2% | -3.1% | 11.4% | 10.4% | -7.7% | 12.4% | 9.9% | 108 |
| **C18:2** | 50-1200 | 50 | 3.3% | 34.8% | 28.0% | 2.5% | 15.0% | 11.4% | 0.2% | 10.0% | 7.6% | 0.1% | 10.4% | 7.8% | -7.8% | 15.6% | 12.1% | 124 |
| **C18:1** | 50-1200 | 50 | 10.3% | 15.7% | 16.5% | 3.7% | 9.7% | 12.1% | -11.9% | 6.5% | 4.9% | -2.6% | 10.0% | 9.2% | -2.5% | 10.5% | 8.2% | 111 |
| **C18:0** | 50-1200 | 50 | 70.3% | 47.4% | 71.0% | -5.8% | 44.4% | 56.7% | -4.8% | 6.1% | 5.4% | -4.4% | 5.4% | 4.4% | -9.1% | 5.2% | 5.9% | 112 |
| **C19:0** | 50-1200 | 50 | 26.9% | 21.7% | 18.1% | 12.4% | 12.2% | 9.6% | -7.7% | 5.8% | 5.4% | -2.8% | 3.8% | 2.9% | -4.7% | 5.9% | 4.6% | 115 |
| **C20:5** | 50-1200 | 50 | -5.5% | 9.3% | 9.0% | -3.4% | 21.9% | 18.2% | 12.8% | 4.8% | 4.8% | -1.8% | 3.3% | 6.7% | -19.0% | 10.2% | 19.0% | 115 |
| **C20:4** | 50-1200 | 50 | -63.6% | 39.4% | 65.3% | 2.3% | 9.2% | 8.3% | 3.8% | 8.3% | 8.4% | 2.7% | 4.6% | 5.5% | -7.7% | 6.8% | 7.9% | 92 |
| **C20:3** | 50-1200 | 50 | -65.6% | 37.9% | 59.2% | 4.2% | 9.3% | 10.0% | 3.5% | 8.3% | 8.3% | 2.3% | 5.1% | 5.8% | -8.9% | 6.9% | 7.7% | 96 |
| **C20:1** | 50-1200 | 50 | -57.4% | 33.5% | 46.0% | 1.6% | 6.3% | 5.1% | 3.7% | 8.9% | 8.4% | 2.1% | 4.3% | 5.0% | -7.9% | 6.9% | 7.4% | 98 |
| **C20:0** | 50-1200 | 50 | -52.5% | 49.1% | 47.8% | 0.4% | 8.5% | 6.6% | 5.3% | 8.9% | 8.9% | 4.0% | 6.3% | 6.2% | -6.6% | 6.6% | 6.9% | 100 |
| **C21:0** | 50-1200 | 50 | -53.3% | 57.8% | 52.0% | 14.1% | 11.6% | 8.9% | -8.3% | 7.4% | 7.6% | -6.1% | 5.9% | 6.2% | -14.1% | 7.5% | 7.8% | 84 |
| **C22:6** | 50-1200 | 50 | -27.4% | 17.1% | 13.6% | 2.0% | 6.2% | 5.1% | -5.1% | 2.9% | 2.3% | -2.0% | 2.0% | 1.9% | -11.2% | 2.5% | 1.8% | 107 |
| **C22:5** | 50-1200 | 50 | -40.3% | 27.2% | 24.5% | -2.3% | 5.0% | 3.9% | -6.5% | 3.4% | 2.5% | -2.5% | 2.1% | 2.0% | -12.1% | 1.9% | 1.5% | 104 |
| **C22:1** | 50-1200 | 50 | -39.8% | 20.5% | 17.0% | -1.5% | 4.2% | 3.2% | -5.5% | 3.0% | 2.3% | -1.9% | 2.5% | 2.2% | -10.8% | 1.6% | 1.8% | 105 |
| **C22:0** | 50-1200 | 50 | -33.3% | 24.5% | 20.4% | -4.6% | 7.1% | 5.8% | -3.7% | 3.7% | 2.6% | -1.2% | 3.7% | 3.0% | -9.2% | 2.9% | 2.7% | 109 |
| **C23:0** | 50-1200 | 50 | -43.3% | 38.8% | 29.7% | 2.6% | 9.4% | 7.0% | -9.8% | 4.4% | 3.2% | -5.9% | 3.1% | 2.7% | -13.6% | 2.6% | 2.1% | 98 |
| **C24:1** | 50-1200 | 50 | -42.8% | 23.1% | 17.2% | -4.7% | 9.2% | 7.1% | -6.6% | 4.0% | 3.2% | -3.4% | 3.7% | 3.2% | -11.6% | 3.1% | 2.6% | 101 |
| **C24:0** | 50-1200 | 50 | 32.0% | 66.7% | 54.1% | 10.8% | 18.7% | 15.1% | -15.5% | 4.7% | 3.9% | -13.3% | 3.9% | 4.1% | -19.1% | 4.6% | 6.1% | 104 |
| **C26:0** | 50-1200 | 50 | -24.3% | 42.5% | 37.3% | -1.3% | 9.2% | 7.4% | -3.7% | 2.3% | 2.3% | -0.3% | 2.9% | 2.9% | -10.3% | 2.9% | 3.0% | 112 |

***Supplementary Figures***


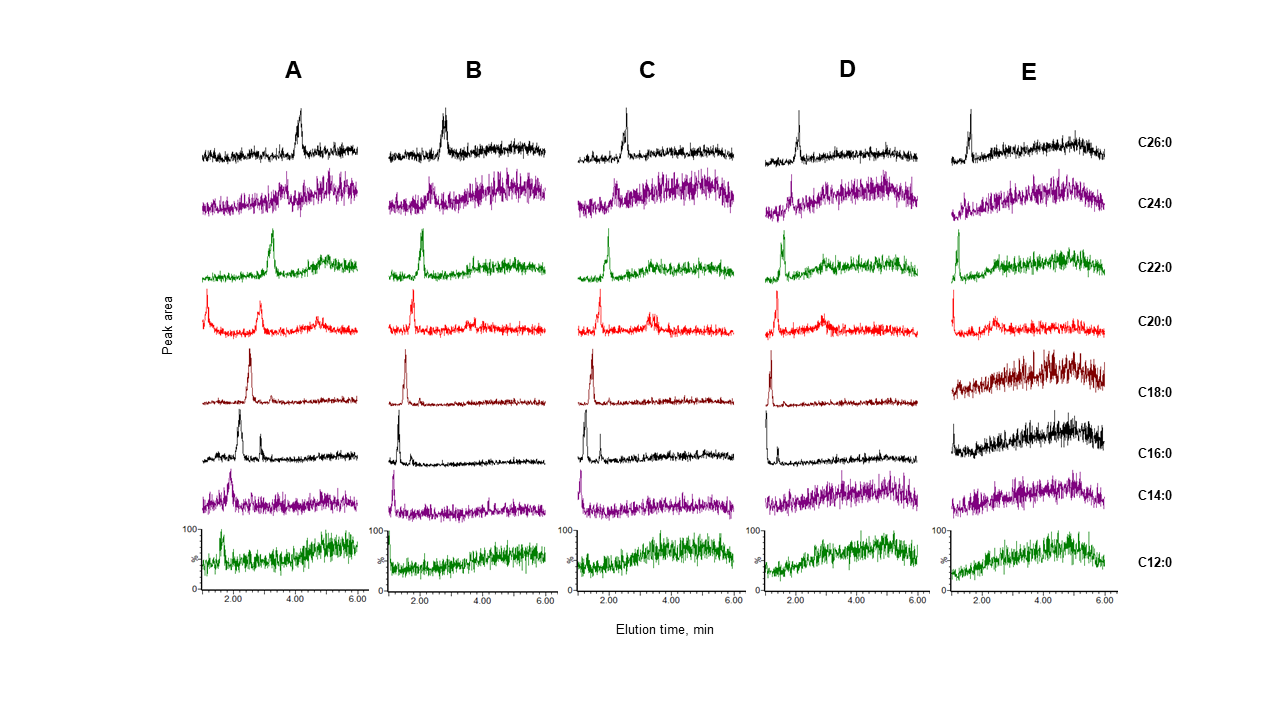


**Fig. S1** Gradient and flow rate optimization for FFA analysis in SFC-MS using (A) 0.6, (B) 0.8, (C) 1.0, (D) 1.2, (E) 1.5 mL/min


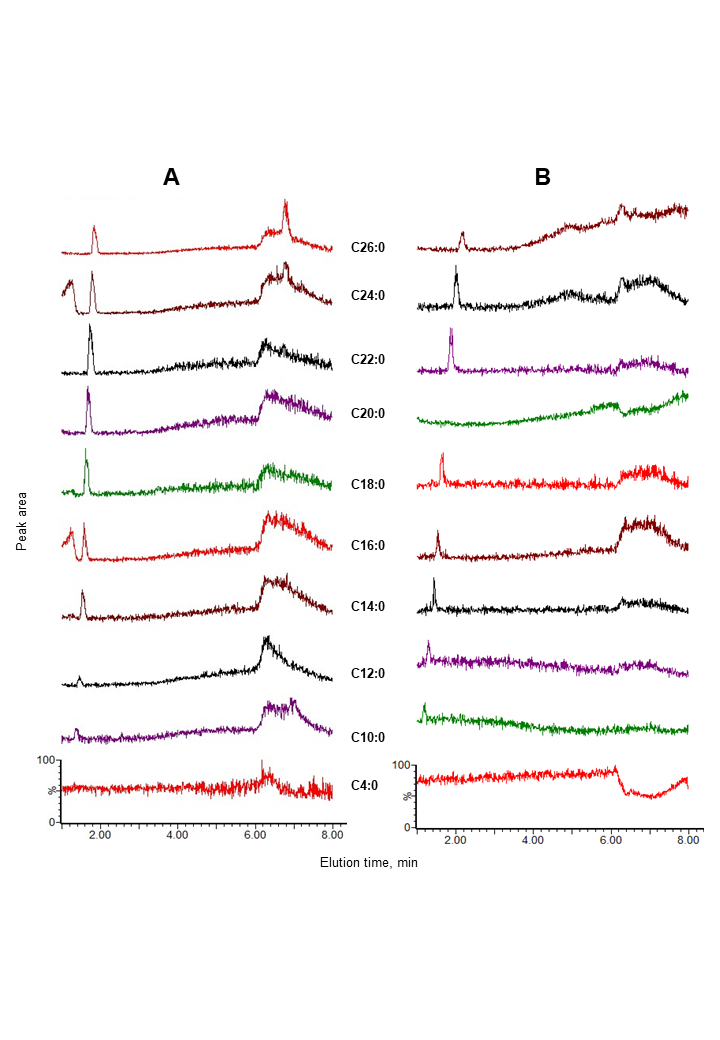


**Fig. S2** Effects of makeup solvent modification in SFC-MS using (A) NH_4_OH and (B) NH_4_F in saturated FFA standards


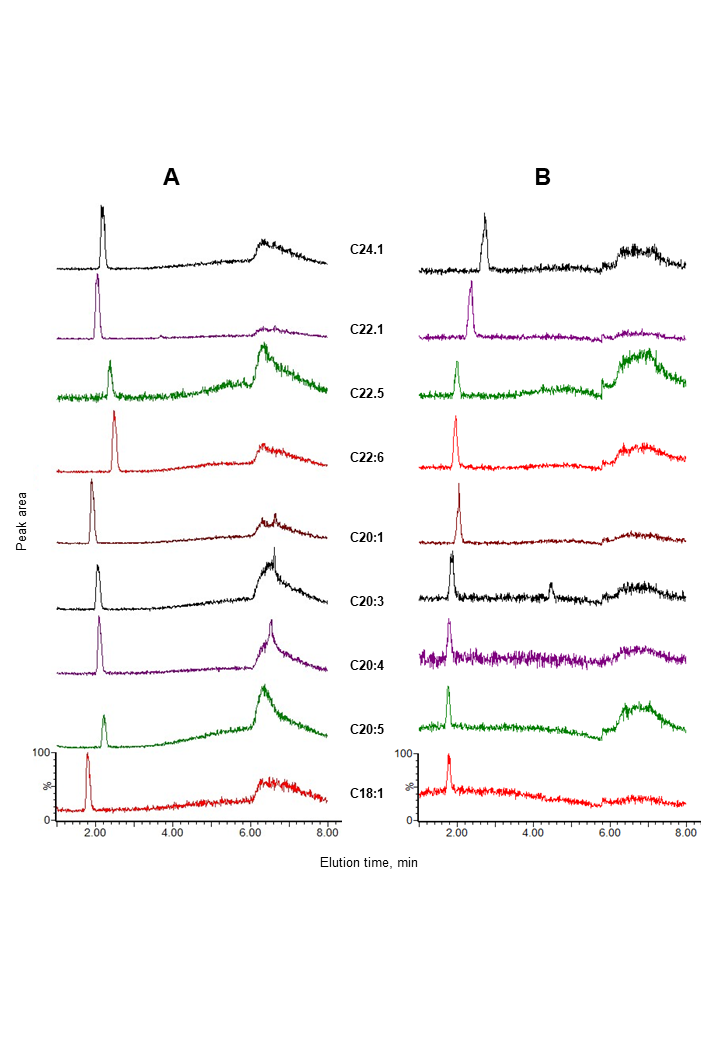


**Fig. S3** Effects of makeup solvent modification in SFC-MS using (A) NH_4_OH and (B) NH_4_F in unsaturated FFA standards


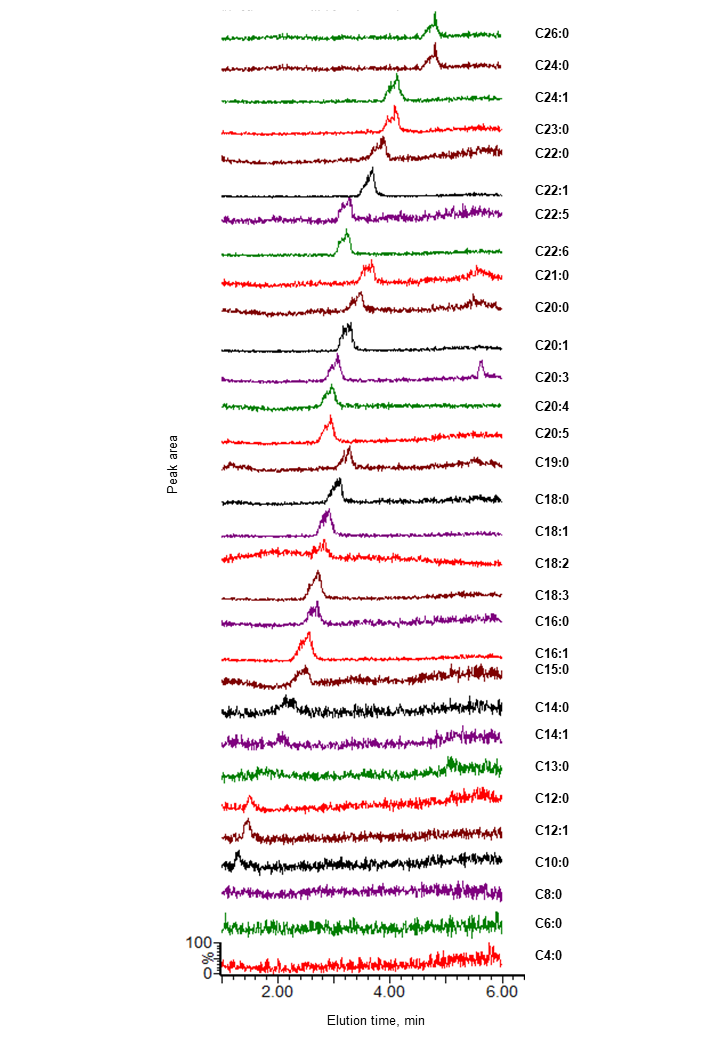


**Fig. S4** SFC-MS overlaid chromatograms of the 31 FFA standards using the Torus 1-AA column


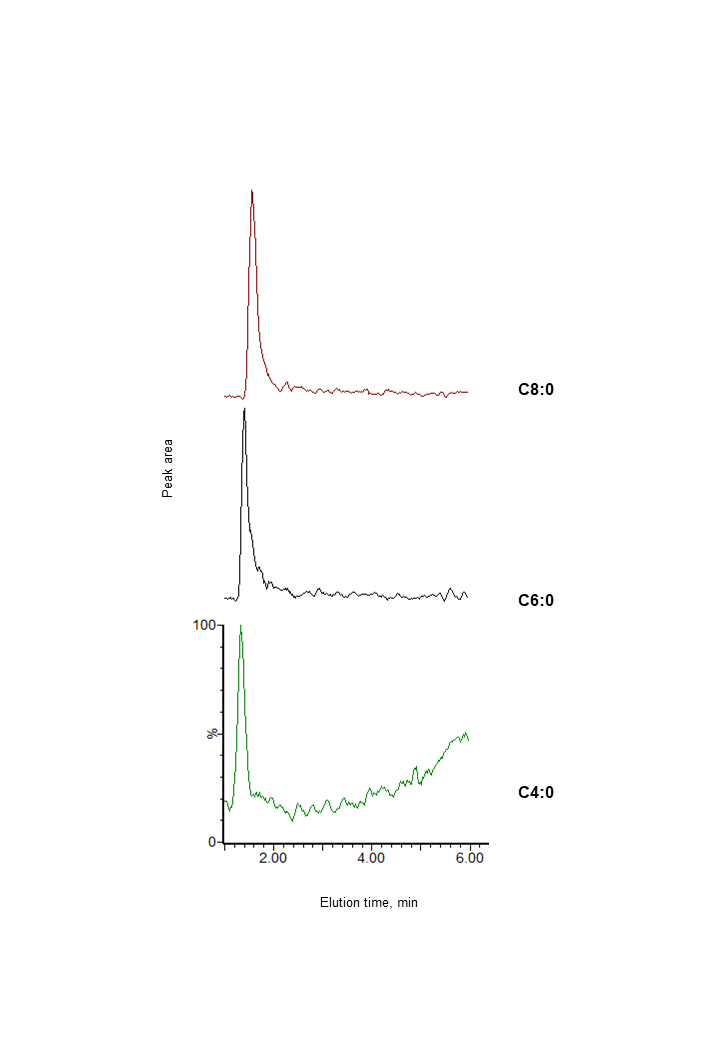


**Fig. S5** SFC-MS detection of SCFFAs (C4:0, C6:0, and C8:0) in the HSS C18 column


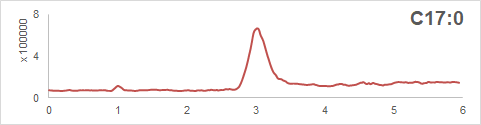


**Fig. S6** Selected ion recording (SIR) chromatogram of heptadecanoic acid, C17:0 as an internal standard for the quantification of pharmaceutical-grade egg yolk powders


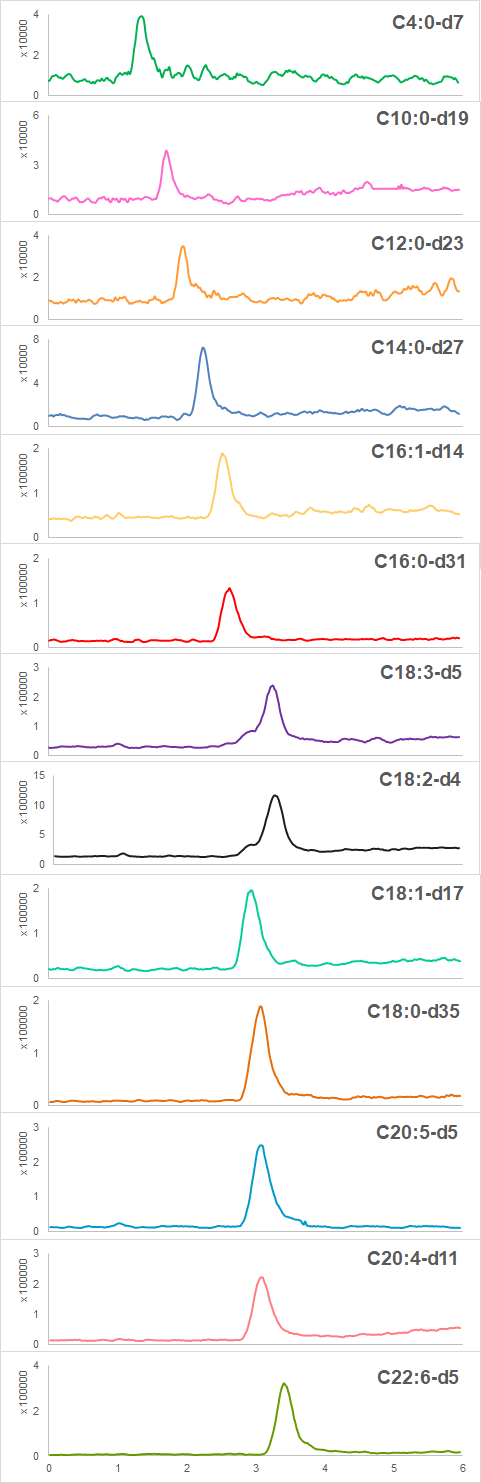


**Fig. S7** Selected ion recording (SIR) chromatograms of 13 deuterated FFAs used as internal standards for the quantification of pharmaceutical-grade egg yolk powders


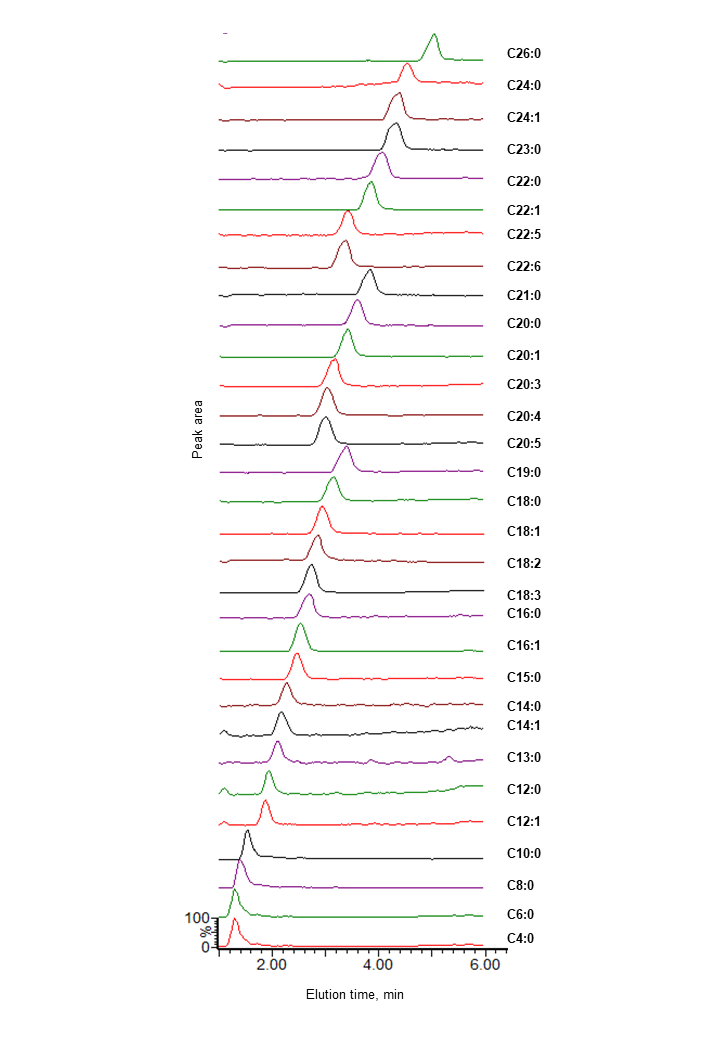


**Fig. S8** SFC-MS overlaid chromatograms of 31 FFA standards using the HSS C18 column


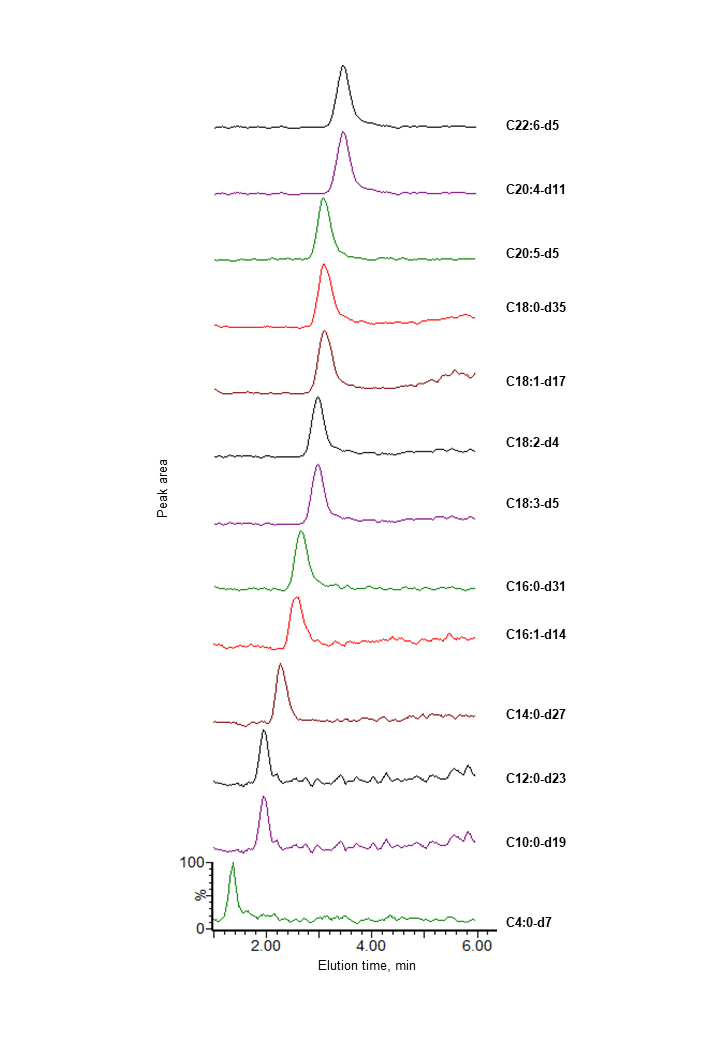


**Fig. S9** SFC-MS overlaid chromatograms of 14 deuterated FFA internal standards using the HSS C18 column


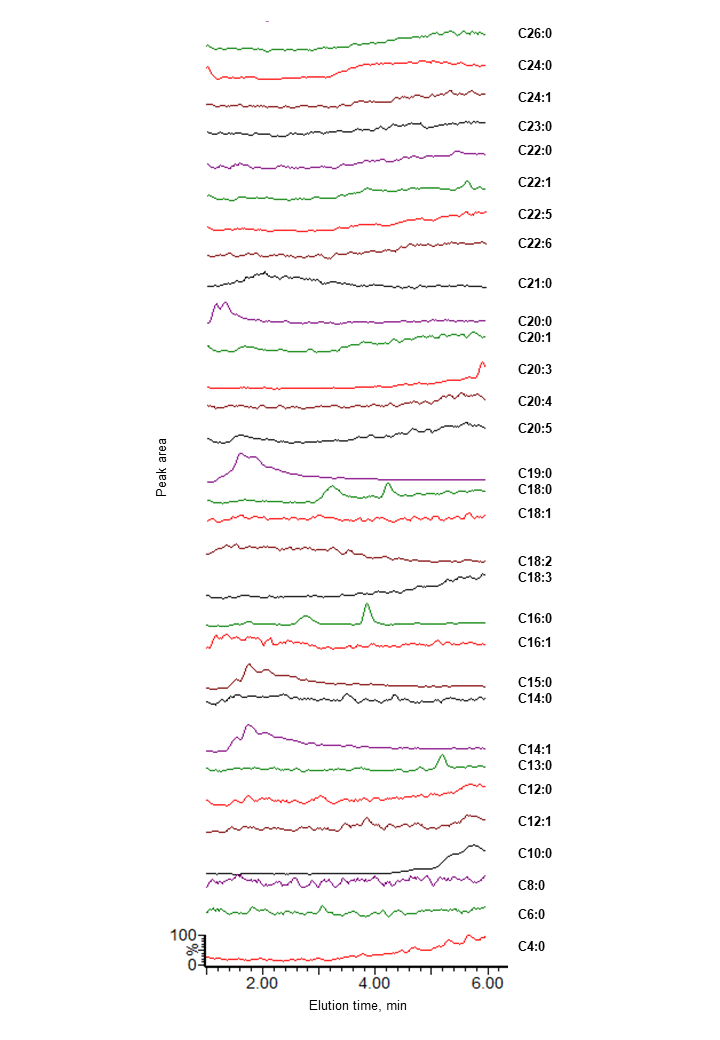


**Fig. S10** Evaluation of systematic carryovers in SFC-MS method in analyzing 31 FFAs


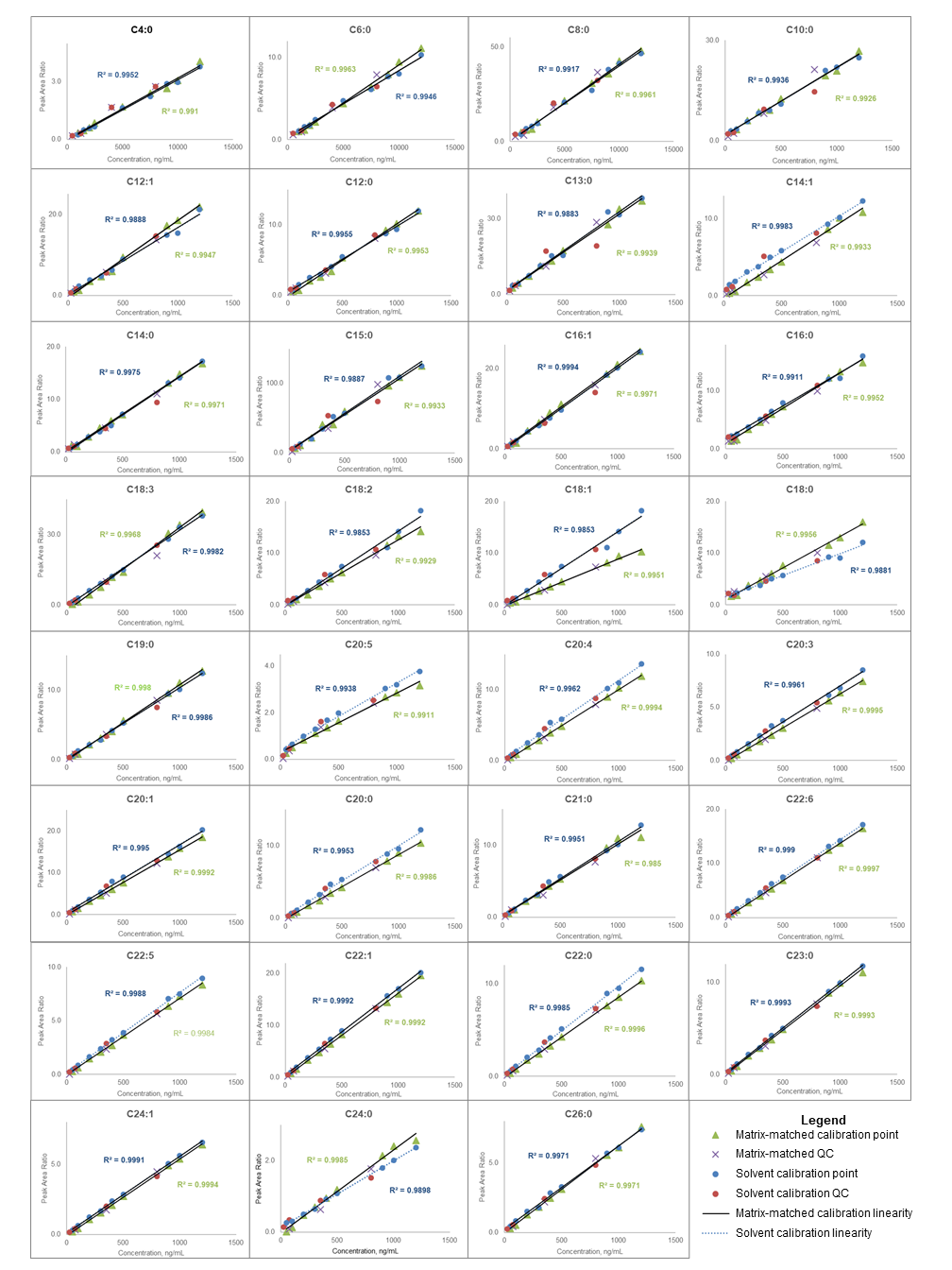


**Fig. S11** Construction of the solvent and matrix-matched calibration curves for 31 FFA standards using the deuterated FFA internal standards
